# Behavioral attenuation of marble burying and digging mirrors evoked and non-evoked phenotypes in the endometriosis mouse model

**DOI:** 10.1038/s41598-026-40662-9

**Published:** 2026-02-20

**Authors:** Samruddhi Deshpande, Roja Barik, Atharvaraj Hande, Richa Patel, Souvik Dey, Amrita Parida, Swar Shah, Rahul Dutta

**Affiliations:** 1 Division of Reproductive Biology, Department of Reproductive Science, Kasturba Medical College, Manipal Academy of Higher Education, Manipal, Karnataka 576104 India; 2 Department of Biotherapeutics Research, Manipal Academy of Higher Education, Manipal, Karnataka 576104 India; 3 Department of Pharmacology, Kasturba Medical College, Manipal Academy of Higher Education, Manipal, Karnataka 576104 India; 4 Krishna Nursing Home, 4-D, Near Delux Bus Stand, Tashkand Society, Nizampura, Vadodara, Gujarat 390002 India

**Keywords:** C57BL/6J, Endometriosis, Behavioral tests, Ethological study, Hyperalgesia, Mice model, Medical research, Neuroscience, Physiology, Zoology

## Abstract

Endometriosis (ENDO) is an estrogen-dependent chronic gynaecological manifestation of uterine fragments attached outside of the uterus, accompanied by debilitating chronic pelvic pain. The evoked assays often employed fail to capture the subtle behavioral nuances in the mouse model. Thus, this study proposes integrating a range of non-evoked assays alongside the ever-so-often used evoked test to better understand ethological disparities in the endometriosis mouse model. In this current study, we developed a syngeneic mouse model using C57BL/6J mice and injected donor uterine fragments into estrogen-primed recipient mice. The endometriotic (ENDO) mice were subjected to behavioral assays like marble burying (MB) and digging. In addition, findings of MB and digging were validated by standard non-evoked and evoked tests to capture the differential and subtle nuances in behavior post-endometriosis induction. The successful ENDO lesion development was confirmed via hematoxylin and eosin staining of lesions, higher M2 macrophage levels from the peritoneal fluid, and higher estrogen levels obtained from ENDO mice. Compared with control mice, ENDO mice exhibited diminished MB (****p value < 0.0001) and digging capacity (****p value < 0.0001). The reduced MB and digging positively correlate with diminished active burrowing, grooming, and exploratory behavior during the open-field test, but with increased anxiety as assessed by the elevated plus maze. The highest positive correlation for MB is with increased anxiety (r = 0.65), and that for digging is with the overnight burrow score (r = 0.62). On the contrary, diminished MB and digging negatively correlate with increased abdominal bouts [(r = -0.44) and (r = -0.46), respectively]. The ENDO mice also showed signs of mechanical and thermal hyperalgesia compared with control mice, as measured by evoked tests. The evaluation of the correlation between MB and evoked tests showed the highest positive correlation with tail flick (r = 0.66) and digging with Von Frey (r = 0.80). The non-evoked tests, such as MB, digging, and grooming patterns, are novel assays to evaluate in the mouse model of ENDO. When incorporated with other non-evoked and evoked tests, it provides a more detailed understanding of ethological behaviors in the ENDO mouse model.

**Supplementary Information:**

The online version contains supplementary material available at 10.1038/s41598-026-40662-9.

## Introduction

Endometriosis (ENDO) is one of the most prevalent reproductive disorders among women. It is characterized by viable endometrial tissue growing in ectopic regions. This gynecological disorder affects 10% of women of reproductive age globally^1^. Common manifestations of ENDO include increased frequencies of dysmenorrhea/chronic pelvic pain (CPP) (40–60%), subfertility (30–50%), and pelvic discomfort (71–87%)^2^. The symptoms impair women’s quality of life and reduce productivity**.** The etiology of ENDO remains unclear, and its manifestations are highly heterogeneous and complex. The existing gaps regarding its pathophysiology make developing a consistent model challenging. ENDO occurs spontaneously only in humans and certain primates. However, due to their cost-effectiveness and experimental flexibility, rodents are most widely used for research and therapeutic testing^3^. High attrition rates and poor translational success from preclinical animal studies frequently limit the development of new analgesics for endometriosis and other pain-related disorders^4^. This may partly stem from the disparity between how pain is measured in nonverbal rodent models and how it is perceived and reported in humans^5^. Another challenge is the lack of an established behavioural endpoint as a surrogate for pain experience^6^. Therefore, successful outcomes in preclinical studies do not necessarily translate into efficacy in clinical trials of pain therapeutics^7^.

Pain assessment in preclinical models primarily relies on eliciting responses to noxious stimuli, such as heat or pricks, and measuring them using nociception assays, such as the hot plate test and Von Frey. The ENDO model is expected to exhibit shorter response time to the applied stimuli. However, these measures do not fully replicate how pain is perceived and assessed in humans. Moreover, evoked tests fail to capture the broader, subtle spontaneous behavioral patterns that hold the key to solving the puzzle of ENDO-induced pain. These traditional assays overlook crucial affective symptoms like apathy and fatigue symptoms that directly correspond to the significant morbidity associated with chronic pain states^8^. ENDO patients suffer from a wide range of physical and psychological symptoms restricting their ability to lead an everyday life^9^. Stimulus-free (non-evoked) behavioral assessments in animal models are more relevant to the patient experience^10^. These assessments for ENDO include three main behaviours: abdominal squashing, contortions, and licking^6^. Additionally, ethological assays measuring the natural well-being of animals, such as nest building^11^, burrowing^12^, locomotion, and thigmotaxis^13–15^, have gained popularity in recent years. These non-evoked measures may correlate better with intrinsic pain and are therefore more relevant to the chronic pain symptoms experienced by individuals with endometriosis.

In response to this translational gap, recent guidelines, including the WERF EPHect-EM-assays, aim to thoroughly analyze and comprehensively decode the complex, multidimensional experience of ENDO-related pain^6^. Home-environment-based approaches represent a substantial refinement of the use of animals in pain research. These assays capture innate, species-typical behaviors that reflect the animal’s overall well-being, motivation, and affective state in a non-induced setting. Behaviors that require sustained effort and high motivation, such as burrowing, are known to be altered in models of chronic inflammation and neuropathic pain^12^. Digging is another such ethological behaviour that can be used to assess an animal’s overall well-being. This behavior reflects the natural inquisitiveness of mice as they explore an open, uniform environment with a compacted digging substrate^16^. Digging and marble-burying (MB) are species-typical behaviors known to be influenced by factors such as species differences, strain variation, hippocampal lesions, and various experimental treatments^17,18^.

Compared to burrowing, digging is more straightforward to assess and less goal-directed, thereby reducing potential confounding from an animal’s motivation to engage in the behavior. Therefore, we aimed to determine whether endometriosis-associated chronic pain leads to measurable deficits in motivated, ethologically relevant behaviors such as digging and MB. This study is the first to use MB and digging assays to characterize a robust motivational-deficit phenotype in a validated non-surgical mouse model of endometriosis. By integrating a comprehensive panel of non-evoked assays with conventional evoked tests, our work addresses the critical need for translationally relevant endpoints in preclinical pain research.

## Materials and methods

### Animal maintenance

Adult C57BL/6J female mice (8–9 weeks old, weighing 20–30 g were procured from the Central Animal Research Facility at Manipal Academy of Higher Education. They were housed under standard conditions of controlled temperature (23 ± 2 °C), humidity (50–55%), and a light–dark cycle (12-12 h), with ad libitum access to water and food throughout the study. Animals were group-housed (4 per cage) in ventilated polypropylene cages with husk bedding. The study was approved by the Institutional Animal Ethics Committee at Kasturba Medical College, Manipal (Approval Number: C57BL/6J- IAEC/KMC/20/2025). All animal experiments were conducted following the Institutional guidelines and regulatory standards in compliance with ARRIVE guidelines for the care and use of laboratory animals.

### Experimental design and timeline

**Fig. 1:**
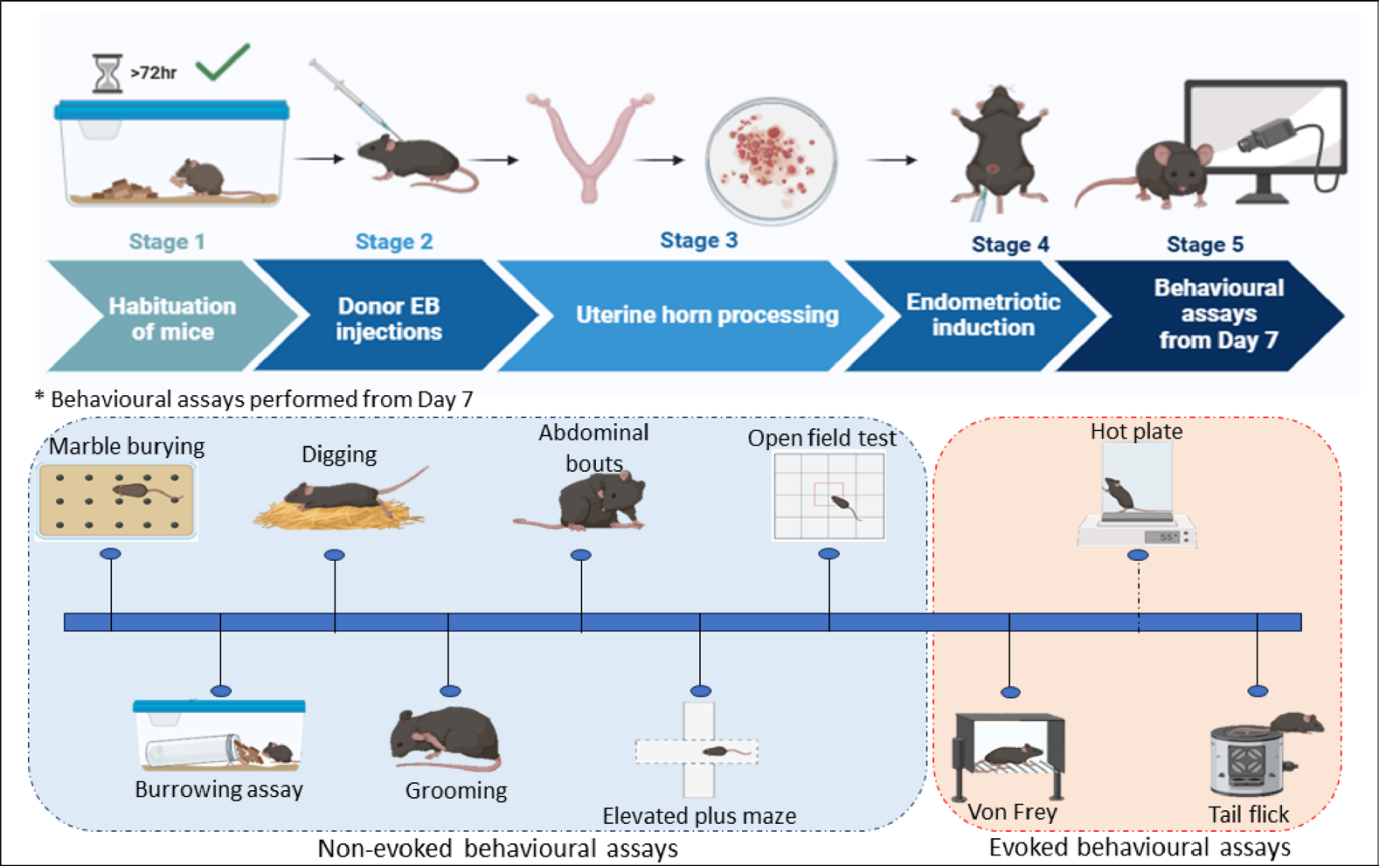
Experimental timeline for syngeneic ENDO mice model and behavioural assessment.

We conducted a methodological study using C57BL/6 J mice, with 53 mice in total (14 controls, 13 donors, and 26 recipients). We developed a syngeneic mouse model as described in Fig. 1. The average weight at the time of sacrifice for control (26.99 g ± 2.82) and ENDO (27.39 g ± 2.64) was recorded, respectively. For blood and peritoneal fluid collection, the mice were anesthetized with an injection of 0.1 mL per 10 g of body weight of Xylazine and Ketamine (100 mg/kg Ketamine, 16 mg/kg Xylazine). Following anesthesia, PBS was infused into the abdominal cavity to collect peritoneal fluid, which was processed for flow cytometry. Blood was collected via cardiac puncture under anesthesia. Serum was separated by centrifugation at 3000 × g for 10 min at 4 °C and stored at -80 °C for ELISA. The ENDO mice were surgically dissected to remove lesions. The ectopic lesions were photographed for documentation, fixed in Bouin’s solution for 24 h, and then transferred to 70% ethanol for histological analysis. The overview of the experimental design is depicted in Fig. 1.

The donor, control, and recipient mice were allowed to acclimate for 3 days before the experimental procedure. The donor animals were primed with estradiol benzoate (EB) (TCI chemicals, #E0329) subcutaneously (s.c.) at 3 μg/mouse for 7 days to stimulate endometrial growth. Then, the donor mice were sacrificed, uterine horns (UH) were extracted, washed with sterile 1 × Phosphate Buffer Saline (1 × PBS) containing penicillin (100 U/mL) and streptomycin (100 mg/mL) (Pen/strep, Thermo Fisher Scientific #15,140,122), split longitudinally and minced into small cell aggregation suspension of UF (< 0.1 mm) consisted of both eutopic endometrium and uterine muscle. They were then reconstituted in 0.5 mL of 1 × PBS without pen/strep, passed through an 18-gauge needle (Dispovan #45,678) to ensure consistent UF size (approximately 1 mm), and then i.p. injected into two recipient mice. By this process, each mouse receives endometrium originating from half a uterus. Mice in the control group were injected with EB (s.c.) (3 µg/mouse) and 1 × PBS (i.p.) at the same dose and time points as recipients. To limit fluctuations and maintain steady estrogen levels, the recipient animals were primed with a single dose of EB (3 µg/mouse) before the UF injection to synchronize the estrous cycle. All recipients received a dose of EB (3 µg/mouse) every 3 days until sacrifice to maintain circulating estrogen. On day 7 post-induction, the control and ENDO mice are subjected to non-evoked assays, such as marble burying, digging, BA, grooming, abdominal bout assessment, EPM, and OFT, and to evoked tests, such as Von Frey, hot plate, and tail flick. The mice were sacrificed on Day 12 post-induction and observed for lesion occurrence.

### Development of a syngeneic ENDO mouse model

The approach followed the ENDO induction protocol we previously established^19^. The detailed methodology of ENDO induction is described in Fig. 1. Briefly, donors and recipient mice were acclimated for 3 days before the experimental procedure, during which the estrous cycle stages were monitored. Upon confirmation of the mice’s daily cycling, they were randomly assigned to the control, donor, or ENDO group. The donor animals were primed with estradiol benzoate (EB) (TCI chemicals, #E0329) subcutaneously (s.c.) at 3 μg/mouse for 7 days to stimulate endometrial growth. Then, the donor mice were sacrificed, uterine horns (UH) were extracted, washed with sterile 1 × Phosphate Buffer Saline (1 × PBS) containing penicillin (100 U/mL) and streptomycin (100 mg/mL) (Pen/strep, Thermo Fisher Scientific #15,140,122), split longitudinally and minced into small cell aggregation suspension of UF (< 0.1 mm) consisted of both eutopic endometrium and uterine muscle. They were then reconstituted in 0.5 mL of 1 × PBS without pen/strep, passed through an 18-gauge needle (Dispovan #45,678) to ensure consistent UF size (approximately 1 mm), and then i.p. injected into two recipient mice. By this process, each mouse receives endometrium originating from half a uterus. Mice in the control group were injected with EB (s.c.) (3 µg/mouse) and 1 × PBS (i.p) at the same dose and time points as recipients. Post-ENDO, all recipient mice were returned to their home cages, and their health was observed for the next 12 days with respect to food and water intake. To limit fluctuations and maintain steady estrogen levels, the recipient animals were primed with a single dose of EB (3 µg/mouse) before the UF injection to synchronize the estrous cycle. All recipients received a dose of EB (3 µg/mouse) every 3 days until sacrifice to maintain circulating estrogen.

### Behavior assessment using non-evoked tests

Behavioral assays provide vital insights into the presentation of anxiety/pain in animal models of ENDO. The assessment of behavioral patterns involved complementing non-evoked (non-induced/natural) behavioral patterns with commonly employed evoked tests. All behavioral assessments were conducted between 9:00 AM and 4:00 PM, while BA was performed between 4:00 PM and 6:00 PM for a 2-h timepoint and overnight during the dark cycle (6:00 PM–9:00 AM). All behavioral testing was performed by an investigator blinded to experimental groups. To prevent the sensitivity and stress induced by the handling from skewing results, we adhered to a strict hierarchy of testing, moving from least invasive to most invasive.

Days 7–10 (Low Stress): We prioritized spontaneous, non-evoked assays (e.g., Marble Burying, Digging, Open Field). These tests rely on natural behaviors and involve minimal handling.

Days 11–12 (Higher Stress): Evoked nociceptive tests, which involve direct handling and stimulus application, were performed last.

General acclimation to the facility occurred during the habituation phase (Stage 1 in Fig. 1) before any induction procedures. For the specific behavioral tests (Marble Burying, Digging, Burrowing), the training described refers to habituation to the testing apparatus, not operant learning. Apparatus habituation was conducted immediately before the testing window. The timings varied from test to test (details given under individual tests).

To ensure uniformity and prevent bias, the same investigator performed all measurements in a blinded manner. After testing, mice were returned to their original home cage.

#### Marble burying assay

The MB test was performed as previously described^20^. Mice were acclimated to the behavioral testing room and; training sessions were conducted to help the mice become familiar with the setup by placing them individually in a standard polycarbonate cage (30 X 20 X 13 cm) that contained a depth of 5 cm of sterile husk bedding with fifteen equal sized glass marbles (1.5 cm diameter) were evenly spaced in a 3 X 5 grid placed on top. The marbles were washed with mild detergent, cleaned, and dried between animals to avoid olfactory cues. On the day of testing, each mouse was carefully placed in a cage corner to avoid disturbing marble placement, left to explore for 30 min, and recorded by a Logitech C930e webcam. At the end of 30 min, the total number of marbles buried was calculated. The buried marbles were counted, but they were completely invisible and at least two-thirds of the way into the bedding. The marbles buried every 5 min from the start of the session were also counted post-session from recorded videos to avoid any disturbance or influence on the mice’s behavior.

#### Digging assay

The spontaneous digging assay was assessed following the procedure outlined previously^16^. Mice were acclimated to the behavioral testing room, and training sessions were conducted to help them become familiar with the setup by placing each mouse individually in a standard polycarbonate cage (30 × 20 × 13 cm) filled with 5 cm of sterile husk bedding. On the day of testing, a similar setup was made, and the digging pattern of each mouse was evaluated for 30 min. Digging behavior was further analyzed by reviewing video footage recorded by the Logitech Webcam C930e at the end of the study, with all videos blinded. The latency to initiate digging for each mouse and the total number of digging episodes were recorded. Digging was characterized by coordinated movements of forepaws, hind paws, or the nose to disturb the bedding material. It is an energetic disorder of the substrate, using all four limbs, typically beginning with the hind legs planted in a wide stance, followed by quick movements of the forelimbs, and concluding with the hind limbs kicking the substrate backward to create a burrow.

#### Burrowing assay

We adopted an assay standardized by Deacon with minor modifications^21,22^. To brief, both the controls and ENDO recipient mice were housed individually in separate cages. A burrow tube filled with 200 g of chow diet was placed against the wall of a cage with a layer of husk bedding. The water was supplied without additional feed in the cage hopper for the mice, as this could distract them. Considering Deacon’s demonstration that healthy mice increased their burrowing activity during the second trial and then maintained a high level of burrowing, the animals were given time to acclimate to the burrow setup^21^. The animals were first acclimated to the testing environment and the presence of an empty burrowing tube for 30 min to diminish novelty-related exploration. After habituation, a filled burrow tube holding 200 g of chow pellets was placed into the same home cage. We started the test three hours before the dark cycle to reduce circadian variability. The number of pellets displaced (g) was assessed at two time points: 2 h (short-term) and 16 h (overnight). The measurements were obtained during a single, continuous testing phase, with no repeated trials. After the experiment, all the animals were returned to their respective original cages. The 2-h burrowing behavior pattern was recorded using a Logitech webcam C930e, and the video footage was later evaluated for latency to burrow, time spent in the burrow tube, and active entries into the burrow tube.

#### Sucrose splash test (Self-grooming)

The sucrose splash test is a behavioral assay in rodents that measures self-care and motivational behavior and assesses anhedonia (a symptom of depression). The mice were individually placed in a clean, sterile cage and acclimatized for 5 min. The 10% sucrose solution was sprayed onto the dorsal fur of the mice, and the mice were placed in the arena for 5 min. The self-grooming patterns were recorded with a camera, and the latency to groom and the total number of grooming episodes were later noted from the video recordings. Grooming is defined as a sequence of movements directed toward the face, head, body, or tail.

#### Abdominal bouts assessment

The control and ENDO mice were placed individually in the center of an open Plexiglas box with a clear floor, without husk bedding (50 cm × 50 cm × 40 cm), in a highly illuminated room. The box was elevated, and the camera (Logitech webcam C930e) was placed below it to capture the bouts and abdominal licking. The experimental mouse was placed in one corner of the box and permitted to explore for 10 min. During a 10-min session, animals were evaluated on the number of bouts. The data was analyzed later through video recordings. The bout behavior, or abdominal licking pattern, often reflects discomfort from visceral and abdominal pain.

#### Open field test

The control and ENDO mice were placed individually in the center of an open Plexiglas box with a clear floor, without husk bedding (50 cm × 50 cm × 40 cm), in a highly illuminated room. The box is virtually marked into the center zone and the peripheral zones. The experimental mouse was placed in one corner of the box and permitted to explore for 15 min. During a 15-min session, animals were evaluated for the number of central and peripheral entries, time spent in the central and peripheral zones, total distance travelled, mean speed, freezing episodes, and total mobile and immobile time using a video-tracking system (Logitech webcam C930e). The data were analyzed using the ANY-maze 64-bit version 7.48 software (Stoelting, Wood Dale, IL). Mice with higher levels of anxiety tend to spend more time on the periphery and less time in the center area. The total distance traveled and the mean speed were regarded as measures of locomotor activity.

#### Elevated plus maze

The EPM apparatus consists of 4 arms in total (two open arms and two closed arms), all with the exact dimensions, elevated 50 cm above the floor. The open arms are free of walls, while the closed arms have 15 cm walls at their periphery. Each mouse was placed individually at the center of the apparatus, facing one of the open arms, and allowed to explore for 5 min. The activity was recorded through a webcam (Logitech webcam C930e). The recorded video was analyzed using the ANY-maze 64-bit version 7.48 software (Stoelting, Wood Dale, IL) to determine the number of entries into and time spent in the open and closed arms.

### Behavior assessment using evoked test

#### Automated Von Frey

To assess mechanical hyperalgesia, a von Frey filament method was used (Dynamic Plantar Aesthesiometer, Ugo Basile) according to the methods of Barrot and Gregory.^23^. Mechanical allodynia was examined as an average of measurements taken on day 11 following ENDO induction. For this procedure, calibrated von Frey filaments (Ugo Basile S.R.L.) with a gradually increasing force of 10 g were applied to the abdominal surface as described previously^24^. During the experiments, the mice were placed in individual Plexiglas chambers on top of a wire-meshed floor on the laboratory bench. They were acclimated to the setting for 1 h until exploratory reactions were halted. The calibrated filaments were applied to the abdomen, and the force was gradually and linearly increased. A definite withdrawal response, such as instant abdomen retraction, licking, or flinching, and the corresponding filament force applied (grams) and the time (seconds) were documented as the withdrawal threshold. Intervals of at least 30 min are maintained between applications to prevent mechanical stimulation-induced sensitivity.

#### Hot plate

The hot plate test was conducted by using a commercially available Hot/Cold Plate instrument (Model 35,100, Ugo Basile) and performed as described previously^25^. Mice were allowed to acclimate to the testing room for 30 min before the test. The device consists of a temperature-regulated metal surface, which was heated to a constant temperature of 55.0 °C ± 0.5 °C for heat stimuli. The nociceptive response, or latency to respond to thermal stimuli, is defined as the time (in seconds) from the moment the mouse is placed in the cylinder until it first licks its hind paws, is startled, or jumps off the hot plate surface. Each mouse was tested once per session to avoid thermal burns and substantial latency alterations from repeated assessments.

#### Tail flick (Hargreaves)

The tail flick test was conducted by using a commercially available I.R. Heat-Flux Radiometer instrument (Model 37,300, Ugo Basile) and performed as described previously^26^. Mice were allowed to acclimate to the testing room for 30 min before the test. The device consists of a pinhole through which infrared radiant heat can pass, and the IT intensity was set at 30 Hz. The mice were scruffed, and their tails were placed through the pinhole. The nociceptive response or latency to respond to thermal stimuli is defined as the time (in seconds) taken from the moment it flicks its tail away from the pinhole. Each mouse was tested once per session to avoid thermal burns and substantial latency alterations from repeated assessments.

### Characterization of the syngeneic mouse model of ENDO

#### Hematoxylin and eosin (H&E) staining

We used H&E staining to assess lesion structure, as ENDO is diagnosed by the presence of viable endometrial-like glands and stroma outside the uterus. Briefly, sections (5 µm) placed on slides covered in Poly-L-lysine (PathnSitu Biotechnologies #PS011) were deparaffinized in xylene, rehydrated in descending grades of ethanol, stained with eosin Y (Sigma-Aldrich, #1.15935), followed by haematoxylin (Sigma-Aldrich, #HX03021349), and mounted using DPX mountant. A bright-field microscope (Nikon Eclipse Ei 4W, Nikon, Tokyo, Japan) was used to view the slides, and photos of representative areas were taken. The samples that did not reflect the morphology of the endometrium were not considered for any further investigation.

#### Flow Cytometry (FC)

The PF was collected from the control and the ENDO mice in individual tubes. The mixture was treated with RBC lysis buffer (eBioscience™ 1X RBC Lysis Buffer #00–4333-57) for 15 min, then centrifuged to remove erythrocytes. The cells were subsequently resuspended in DPBS for further analysis. A total of 1 × 10^6^ cells were seeded into each tube after cell counting, then incubated with anti-MO-CD206-PE (eBioscience, #12–2061-80) for measuring M2 macrophages at a concentration of 0.5 µg/test for 30 min at RT in the dark. The cells were then centrifuged to remove unbound antibody, and the pellet was resuspended in 100 µl of DPBS for FC analysis using a BD Accuri™ C6 Plus flow cytometer. The live and single cells were gated in the unstained samples and used as a gating strategy for subsequent samples. Colour compensation for the samples was performed in FlowJo to determine the percentage of positive cells in each channel relative to the unstained sample. (Table 2)

#### Estrogen ELISA

Serum estradiol (E2) concentrations were measured using a commercially available Mouse Estrogen ELISA kit (ELK Biotechnology Co., Ltd., Wuhan, China; Cat. No. #ELK8407) according to the manufacturer’s instructions. Absorbance was measured at 450 nm using a MultiSkan FC Microplate Photometer (Thermo Fisher Scientific, Waltham, MA, USA) equipped with SkanIt software. E2 concentrations (pg/ml) were determined by interpolating the optical density (OD) values into a standard curve.

### Statistical analysis, linear regression, and correlation matrix

Statistical analysis and visualization were performed using GraphPad Prism 8.0.1 (GraphPad Inc., USA). Given the inherent variability and skewed nature of ethological and nociceptive behavioral measures, normality was systematically assessed prior to statistical analysis, as demonstrated in Table 1. When either group failed normality testing, nonparametric analyses such as the Mann–Whitney test (U value) and the p-value obtained were selected to avoid violations of parametric assumptions and to ensure a conservative, biologically meaningful interpretation of behavioral outcomes. Student’s t-tests or one-way ANOVA (Analysis of Variance) were used to compare the means associated with the groups with normal distribution in study parameters. All graphs presented are plotted as the means with standard errors of the means (SEMs). Statistical significance for all tests was established at P < 0.05 for data that met the assumptions for parametric analyses. Significance levels were as follows: * P < 0.05, ** P < 0.01, *** P < 0.001, **** P < 0.0001. The linear regression analysis was performed using GraphPad Prism 8.0.1 (GraphPad Inc., USA), and a correlation matrix was generated using the “corrplot” function in RStudio (RStudio 2023.09.1).

**Table 1:** Normality assessment and non-parametric statistical analysis of behavioral tests. ns = non-significant, *p value < 0.05, **p value < 0.01, ***p value < 0.001, ****p value < 0.0001 represent statistically significant values.

Sr. No	Test parameter	Normal distribution (Yes/No)	Exact p-value	Mann–Whitney score (U)
1	Total marbles buried at 30 min	No	< 0.0001****	16
2	Digging latency	No	0.1183 (ns)	129
3	Digging episodes	Yes	Unpaired t-test	Unpaired t-test
4	Overnight burrow score	Yes	Unpaired t-test	Unpaired t-test
5	2-h burrow score	No	0.0002***	56.5
6	Active burrow entries	No	0.0004***	62
7	Time spent in burrow tube	Yes	Unpaired t-test	Unpaired t-test
8	Latency to burrow	No	0.1603 (ns)	132
9	Grooming latency	No	0.8610 (ns)	175.5
10	Grooming episodes	Yes	Unpaired t-test	Unpaired t-test
11	Abdominal directed licking	Yes	Unpaired t-test	Unpaired t-test
12	EPM entries in open	Yes	Unpaired t-test	Unpaired t-test
13	% time spent in open	No	0.0003***	61
14	EPM entries in closed	No	0.0030**	80
15	% time spent in closed	Yes	Unpaired t-test	Unpaired t-test
16	Mean speed	Yes	Unpaired t-test	Unpaired t-test
17	Entries in central area	Yes	Unpaired t-test	Unpaired t-test
18	Time spent in central area	No	0.0005***	63
19	Entries in peripheral area	No	0.0096**	92
20	Time spent in peripheral area	No	0.0053**	85.5
21	Von Frey Reaction time	No	< 0.0001****	3
22	Von Frey Force	No	< 0.0001****	6
23	Hot plate	No	< 0.0001****	41
24	Tail flick	No	< 0.0001****	0.5

## Results

### Successful ENDO induction and characterization

**Fig. 2:**
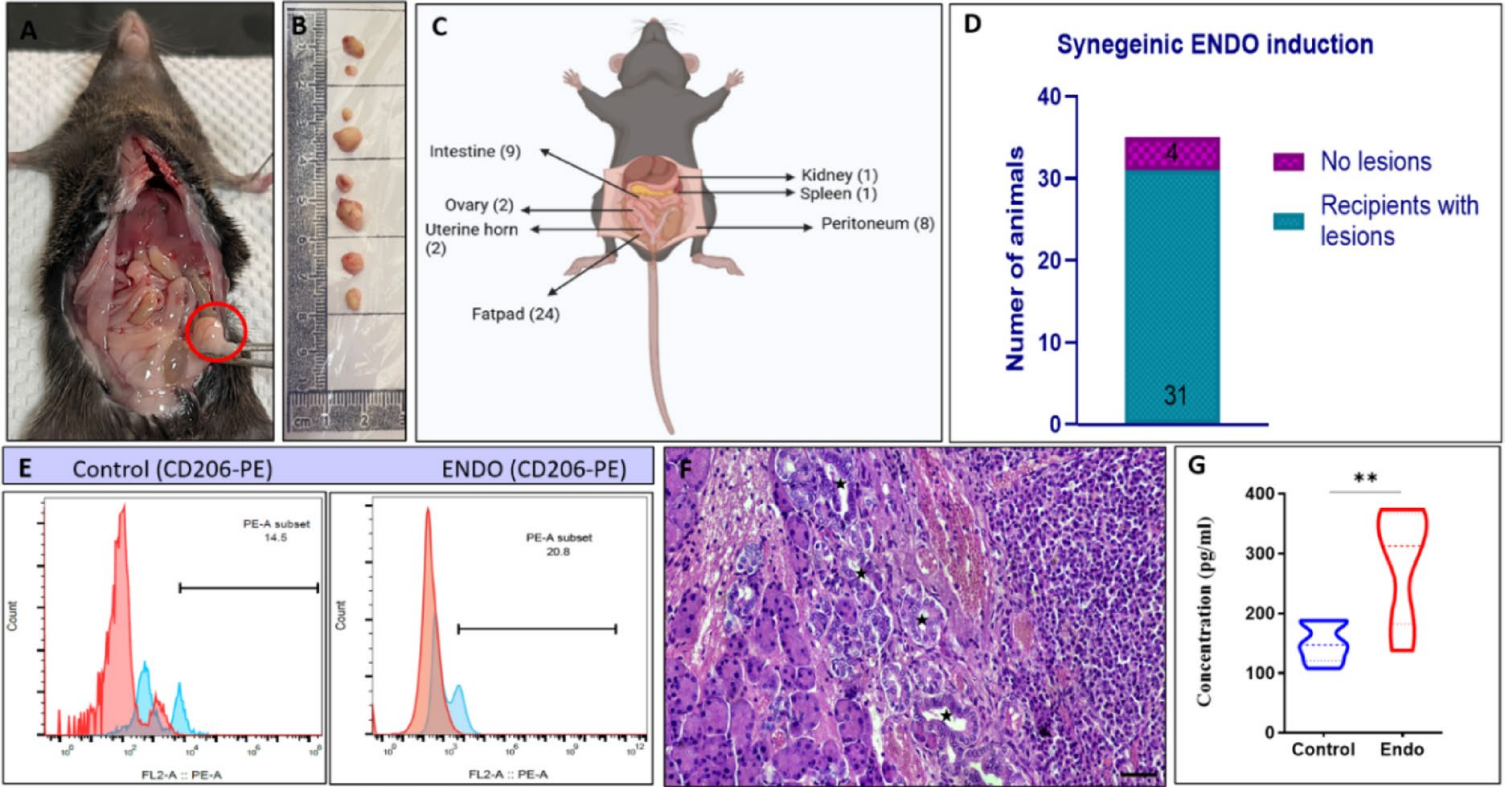
Successful ENDO induction and characterisation: The ENDO induction was performed as shown in Fig. 1. Post-ENDO, all recipient mice were returned to their home cages, and their health was observed for the next 12 days with respect to food and water intake. The ENDO mice were sacrificed after 12 days to assess for ectopic lesions in the peritoneal cavity. (**A**). The representative image of a mouse with an angiogenetic lesion attached to the right fat pad. (**B**). The gross representative images show both white and red lesions obtained from ENDO animals, with the scale bar shown. (**C**). A total of 47 lesions were obtained from 31 ENDO mice, with at least one lesion from each animal. The lesions obtained were mainly adhered to the fat pad (n = 24), intestine (n = 9), peritoneum (n = 8), uterine horn (n = 2), ovary (n = 2), spleen (n = 1), and kidney (n = 1). (**D**). Overall, successful induction was achieved in 31 of 35 ENDO mice, yielding a success rate of 88.57%. (**E**). Representative histogram overlays showing CD206 expression detected using a PE-conjugated anti-CD206 antibody. The x-axis represents PE fluorescence intensity (FL2-A:: PE-A, log scale), corresponding to CD206 expression levels, while the y-axis represents the number of events. Red-filled histograms indicate the negative control population (unstained/isotype control), whereas blue outlined histograms represent CD206-PE–stained cells. A rightward shift of the blue peak relative to the red peak indicates CD206 positivity. The gated PE-A subset shows a higher proportion of CD206-positive cells in the ENDO group (20.8%) than in the Control (14.5%). (**F**). The H & E staining of the lesion obtained from ENDO revealed a characteristic endometriosis signature with multiple glandular epithelium (black star) (Magnification 400x). (**G**). The serum estrogen levels in ENDO mice were significantly higher than in controls.

The representative image of a mouse with a lesion evident of angiogenesis attached to the right fat pad (Fig. 2A). The gross representative images show both white and red lesions obtained from ENDO animals on the scale bar (Fig. 2B). A total of 47 lesions were obtained from 31 ENDO mice, with at least one lesion from each animal. The lesions obtained were mainly adhered to the fatpad (n = 24), intestine (n = 9), peritoneum (n = 8), uterine horn (n = 2), ovary (n = 2), spleen (n = 1), and kidney (n = 1) (Fig. 2C). The overall successful induction was obtained in 31 ENDO mice from a total of 35 induced mice, yielding a successful ENDO induction of 88.57% (Fig. 2D). Representative histogram overlays showing CD206 expression detected using a PE-conjugated anti-CD206 antibody. The x-axis represents PE fluorescence intensity (FL2-A:: PE-A, log scale), corresponding to CD206 expression levels, while the y-axis represents the number of events. Red-filled histograms indicate the negative control population (unstained/isotype control), whereas blue outlined histograms represent CD206-PE–stained cells. A rightward shift of the blue peak relative to the red peak indicates CD206 positivity. The gated PE-A subset shows a higher proportion of CD206-positive cells in the ENDO group (20.8%) than in the Control (14.5%). (Fig. 2E). The lesion, characterized by H & E, revealed a characteristic endometriosis signature with multiple glandular epithelium (black star)(Magnification 400x). (Fig. 2F). The estrogen levels from the serum obtained from ENDO mice revealed a significant increase as compared to the control (Fig. 2G). Out of the 31 ENDO animals, 5 were randomly selected on day 7 for histological and molecular characterization to confirm successful induction, as shown in Fig. 2. All remaining animals were used for behavioral video analysis. Specifically, the results presented are derived from 26 ENDO mice and 14 control animals that underwent comprehensive video tracking and behavioral phenotyping. All animals that underwent video tracking and behavioral assessment are included in the analysis, ensuring no selection bias.

### ENDO mice exhibit reduced MB and digging ethological behavior, and have a weak correlation with lesion number

**Fig. 3:**
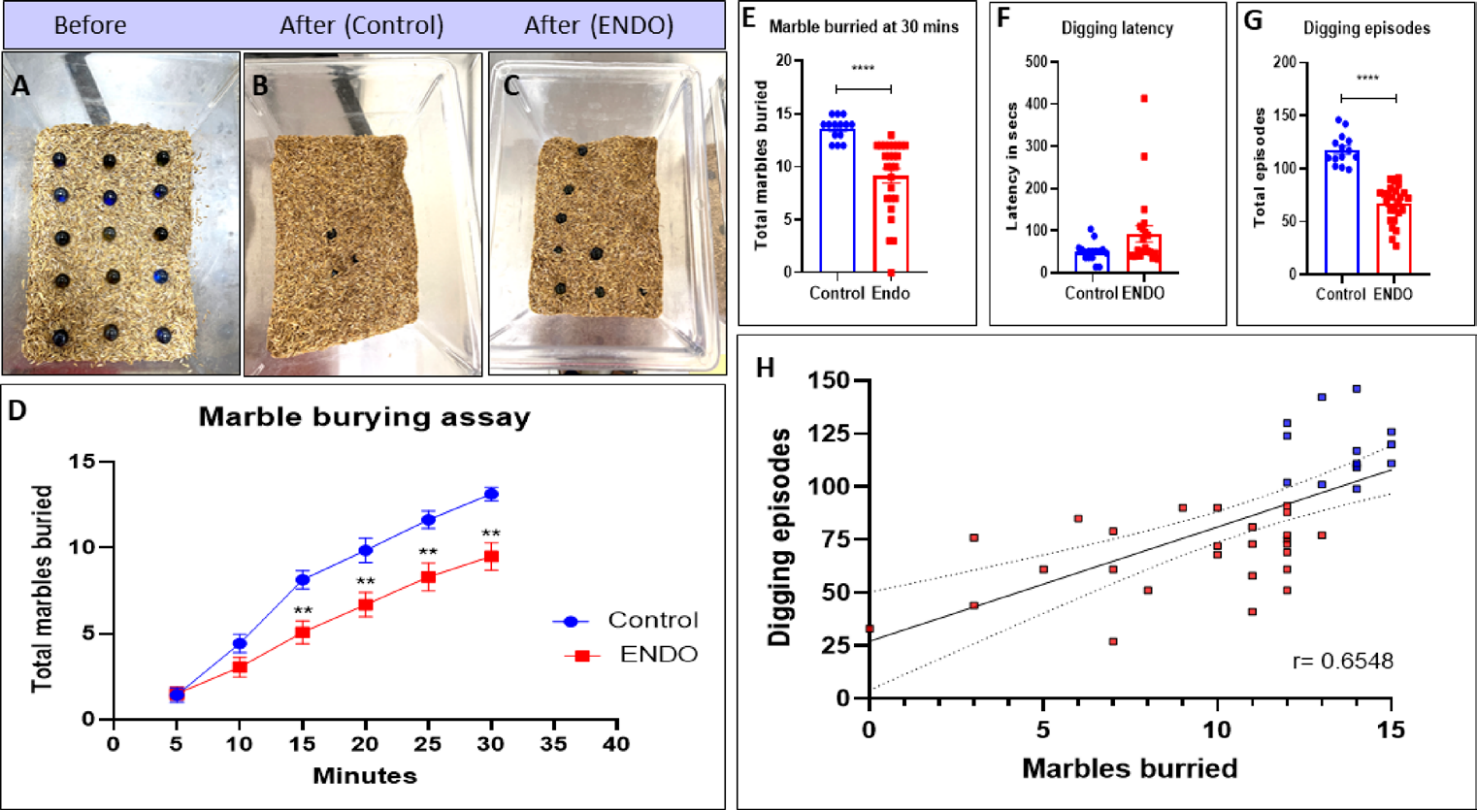
ENDO mice exhibit reduced marble burying and digging ethological behaviour. (**A**, **B**, **C**) Representative images of marble placement before and after the 30-min test showed greater marble burying capacity in the control than in ENDO. (**D**) The marble burying capacity was tracked every 5 min up to the end of the 30-min test, revealing comparable capacity between ENDO (5 min: 1.5; 10 min: 3.038) and control (5 min: 1.429; 10 min: 4.429) at 5-min and 10-min intervals, respectively. Whereas post that there is a significant decline in marble burying capacity between ENDO (15 min: 5.077 (**p value = 0.003); 20 min: 6.692 (**p value < 0.006); 25 min: 8.308 (**p value = 0.007); and 30 min: 9.154 (**p value = 0.002) as compared to control mice(15 min: 8.143; 20 min: 9.857; 25 min: 11.64; and 30 min: 13.64) at 15 min, 20 min, 25 min and 30 min respectively. (**E**) The total marble buried at the end of a 30-min interval revealed a diminished marble burying capacity in ENDO mice (9.154 ± 0.2891) v/s control mice (13.64 ± 0.6764) (****p value < 0.0001, Mann–Whitney U = 16). (**F**) The latency for digging was comparable among ENDO (92.05 ± 19.25) and control mice (49.63 ± 5.633) (p value = 0.1183 (ns), Mann–Whitney U = 129). (**G**) But the overall digging episodes declined in ENDO mice (67.38 ± 3.474) vs. control (117.7 ± 3.892) (**** p value < 0.0001, t value = 9.09). (**H**) Pearson’s correlation analysis between marbles buried and digging episodes revealed a significant, strong positive correlation (r = 0.6548, **** p value < 0.0001). Data represented as mean ± SEM and the t value and p values generated by the unpaired t test from C57BL/6J control, n = 14, and ENDO, n = 26. *p value < 0.05, **p value < 0.01, ***p value < 0.001,****p value < 0.0001 represent statistically significant values.

The representative images of marble placement before and after the post-30-min test showed higher MB capacity in the control than in ENDO (Fig. 3A, B, C). The MB capacity was tracked every 5 min for 30 min, revealing comparable capacity between ENDO (5 min: 1.5; 10 min: 3.038) and control (5 min: 1.429; 10 min: 4.429) at 5-min and 10-min intervals, respectively. Whereas post that there is a significant decline in MB capacity between ENDO (15 min: 5.077 (**p value = 0.003); 20 min: 6.692 (**p value < 0.006); 25 min: 8.308 (**p value = 0.007); and 30 min: 9.154 (**p value = 0.002) as compared to control mice(15 min: 8.143; 20 min: 9.857; 25 min: 11.64; and 30 min: 13.64) at 15 min, 20 min, 25 min and 30 min respectively (Fig. 3D). The total marble buried at the end of a 30-min interval revealed a diminished MB capacity in ENDO mice (9.154 ± 0.2891) v/s control mice (13.64 ± 0.6764) (****p value < 0.0001, Mann–Whitney U = 16) (Fig. 3E). Further, the latency for digging was calculated, and was comparable among ENDO (92.05 ± 19.25) and control mice (49.63 ± 5.633) (p value = 0.1183 (ns), Mann–Whitney U = 129) (Fig. 3F). But the overall digging episodes declined in ENDO mice (67.38 ± 3.474) compared with controls (117.7 ± 3.892) (**** p value < 0.0001, t value = 9.09) (Fig. 3G). Pearson’s correlation analysis between marbles buried and digging episodes revealed a significantly strong positive correlation (r = 0.6548, **** p value < 0.0001) (Fig. 3H). Data represented as mean ± SEM and the t value and p values generated by the unpaired t test from C57BL/6J control, n = 14, and ENDO, n = 26. *p value < 0.05, **p value < 0.01, ***p value < 0.001, ****p value < 0.0001 represent statistically significant values.

**Fig. 4:**
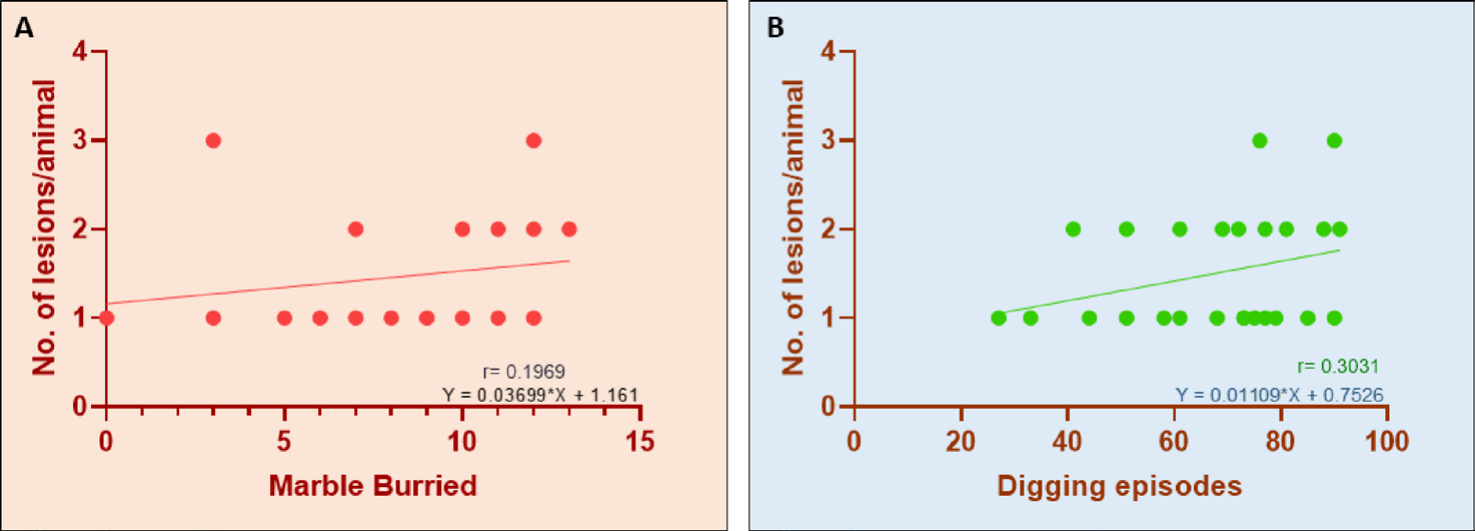
MB and digging show a very weak positive correlation with the number of lesions in ENDO. We evaluated the marble-buried and digging episodes individually, with lesion counts obtained only for each ENDO mouse. (**A**). The correlation and linear regression analysis of marbles buried at the end of 30 min of ENDO in mice with their respective lesion counts showed a very weak positive correlation (r = 0.1969), indicating no significant relationship. (**B**). The correlation and linear regression analysis of digging episodes evaluated over 30 min showed a weak positive correlation (r = 0.3031) with the number of lesions per animal, indicating no significant relationship.

We also evaluated the marble-buried and digging episodes separately, recording the number of lesions only for each ENDO mouse. The correlation and linear regression analysis of marbles buried at the end of 30 min of ENDO mice with their respective number of lesions exhibited a very weak positive correlation (r = 0.1969), indicating no significant relation between them (Fig. 4A). Similarly, the correlation and linear regression analysis of digging episodes evaluated for 30 min indicated a weak positive correlation (r = 0.3031) with number of lesions obtained per animal indicating no significant relation among them (Fig. 4B).

### ENDO mice exhibit diminished burrow score and active burrowing

**Fig. 5:**
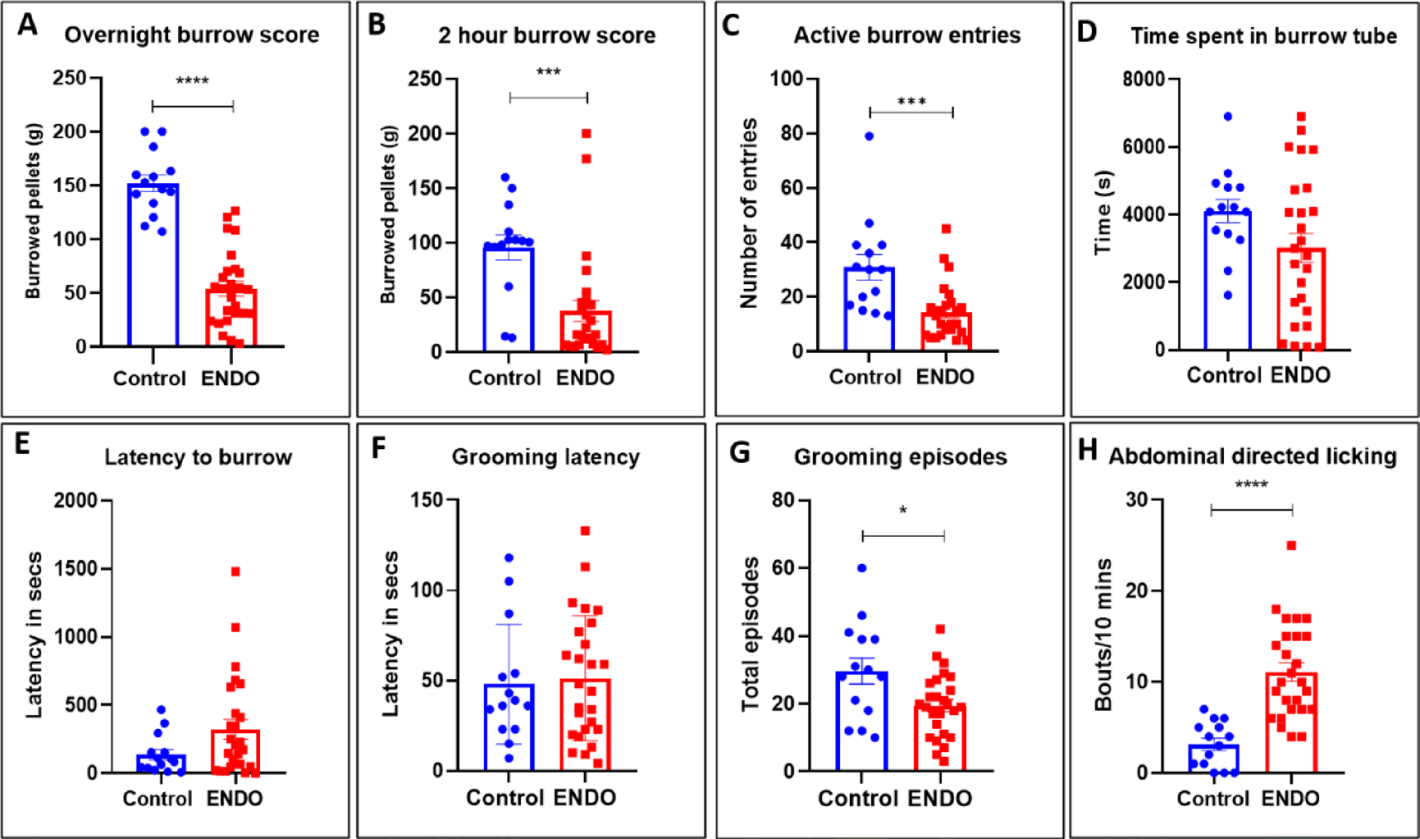
ENDO mice exhibit diminished burrow score, grooming episodes, but increased abdominal bouts: (**A**). The ENDO mice evaluated for BA revealed a reduced burrow score (53.68 g ± 6.76 g) as compared to control (151.8 g ± 7.82 g) (****p value < 0.0001, t value = 9.021). (**B**). The BA assessed at a 2-h interval also showed a similar trend of reduced burrow score in ENDO mice (37.93 g ± 9.773 g) v/s control (95.96 g ± 11.41 g) (***p value = 0.0002, Mann–Whitney U = 56.5). (**C**). The active burrowing, i.e., “active entry of mice in burrow tube,” revealed a reduced number of entries in ENDO mice (14.27 ± 1.961) as compared to control (30.25 ± 5.443) (*** p value = 0.0004, Mann–Whitney U = 62). (**D**). The time spent in the burrow tube was calculated, revealing comparable results in ENDO mice (3019 s ± 424.7) compared to controls (4033 s ± 436.2) (p value = 0.1603 (ns), Mann–Whitney U = 132). (**E**). The latency to burrow was comparable in ENDO mice (321.4 ± 72.29) vs control (134.8 ± 40.84) (p value = 0.0889, t value = 1.747). (**F**). The overall latency to groom was recorded post-sucrose splash test, which revealed comparable results in ENDO (51.23 ± 6.783) vs. control mice (48 ± 8.874) (p value = 0.861 (ns), Mann–Whitney U = 175.5). (**G**). The gross grooming episodes were significantly reduced in ENDO (19.46 ± 1.847) as compared to control (29.75 ± 4.536) (*p value = 0.0164, t value = 2.519). (**H**). The abdominal bouts of mice recorded for 10 min revealed a significant increase in bouts of abdominal licking in ENDO mice (11.12 ± 1.019) as compared to control (3.143 ± 0.6618) (****p value < 0.0001, t value = 5.398). Data represented as mean ± SEM and the t value and p values generated by the unpaired t test from C57BL/6J control, n = 14, and ENDO, n = 26. *p value < 0.05, **p value < 0.01, ***p value < 0.001,****p value < 0.0001 represent statistically significant values.

The overnight BA revealed a reduced burrow score in ENDO mice (53.68 g ± 6.76 g) as compared to control (151.8 g ± 7.82 g) (****p value < 0.0001, t value = 9.021) (Fig. 5A). The BA assessed at a 2-h interval also showed a similar trend of reduced burrow score in ENDO mice (37.93 g ± 9.773 g) v/s control (95.96 g ± 11.41 g) (***p value = 0.0002, Mann–Whitney U = 56.5) (Fig. 5B). The active burrowing, i.e., “active entry of mice in burrow tube,” revealed a reduced number of entries in ENDO mice (14.27 ± 1.961) as compared to control (30.25 ± 5.443) (*** p value = 0.0004, Mann–Whitney U = 62) (Fig. 5C). The time spent in the burrow tube was calculated, revealing comparable results in ENDO mice (3019 s ± 424.7) as compared to control (4033 s ± 436.2) (p value = 0.1647, t value = 1.419) (Fig. 5D). The latency to burrow was comparable in ENDO mice (321.4 s ± 72.29) vs control (134.8 s ± 40.84) (p value = 0.1603 (ns) , Mann–Whitney U = 132) (Fig. 5E). Data represented as mean ± SEM and the t value and p values generated by the unpaired t test from C57BL/6J control, n = 14, and ENDO, n = 26. *p value < 0.05, **p value < 0.01, ***p value < 0.001, ****p value < 0.0001 represent statistically significant values.

### ENDO mice display reduced self-grooming episodes but increased abdominal bouts

The overall latency to groom was recorded post sucrose splash test, which revealed comparable results in ENDO (51.23 ± 6.783) vs control mice (48 ± 8.874) (p value = 0.861 (ns), Mann–Whitney U = 175.5) (Fig. 5F). The gross grooming episodes were significantly reduced in ENDO (19.46 ± 1.847) as compared to control (29.75 ± 4.536) (*p value = 0.0164, t value = 2.519) (Fig. 5G). The abdominal bouts of mice recorded for 10 min revealed a significant increase in bouts of abdominal licking in ENDO mice (11.12 ± 1.019) as compared to control (3.143 ± 0.6618) (****p value < 0.0001, t value = 5.398) (Fig. 5H). Data represented as mean ± SEM and the t value and p values generated by the unpaired t test from C57BL/6J control, n = 14, and ENDO, n = 26. *p value < 0.05, **p value < 0.01, ****p value < 0.0001 represent statistically significant values.

### ENDO mice display increased anxiety but diminished exploratory potential

**Fig. 6:**
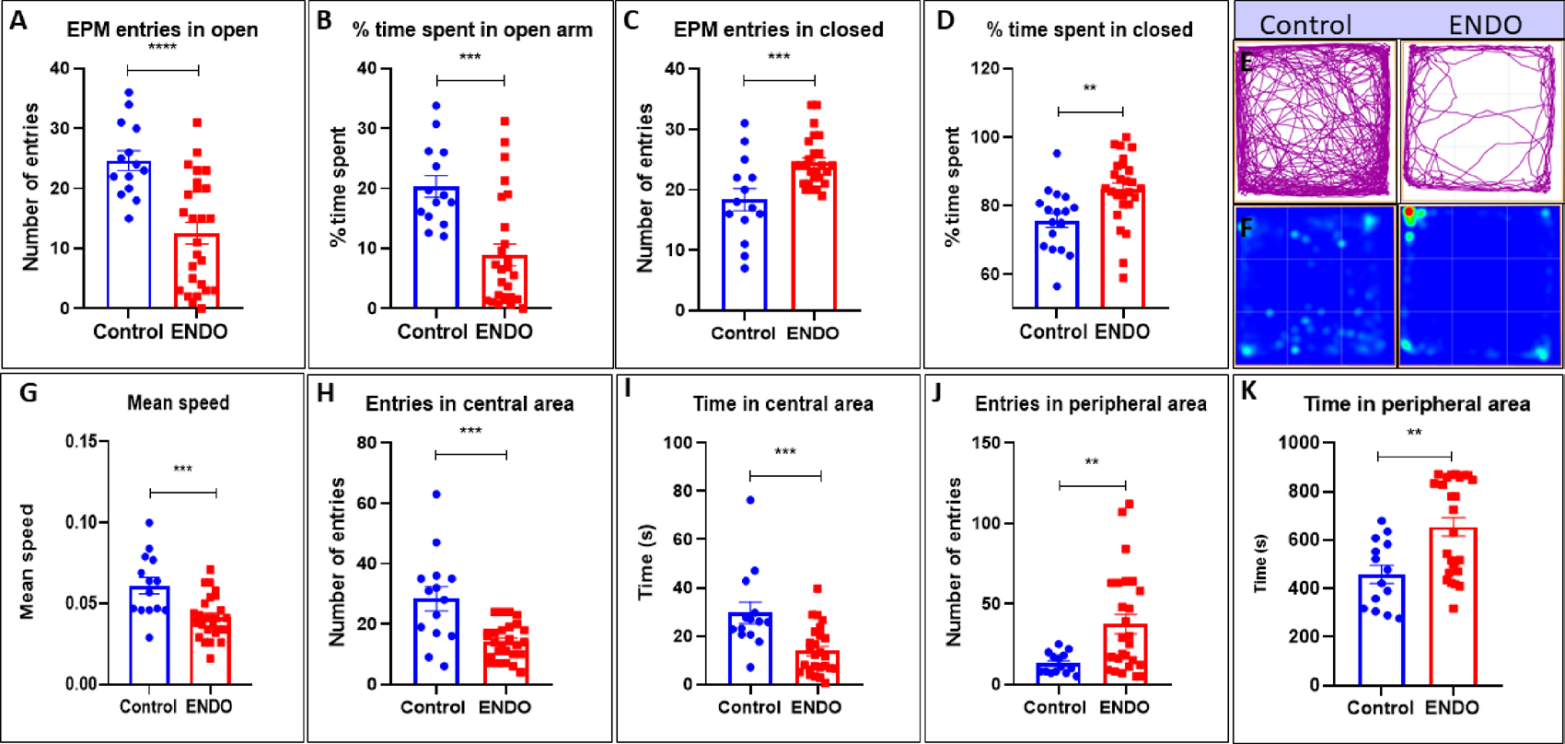
ENDO mice show increased anxiety but diminished exploratory behaviour. (**A**). The anxiety parameters were calculated via EPM revealing significant reduction in entries in open arm in ENDO (12.54 ± 1.801) vs control mice (24.64 ± 1.65) (****p value < 0.0001, t value = 4.411) and (**B**) % time spent in open arm in ENDO (8.936% ± 1.819) vs control mice (20.34% ± 1.81) (***p value = 0.0003, Mann–Whitney U = 61). (**C**) As opposed to significant increase in entries in closed arms in ENDO (24.5 ± 0.8119) vs control mice (18.36 ± 1.848) (**p value = 0.003, Mann–Whitney U = 80) and (**D**) significant increase in % time spent in closed arms in ENDO (84.71 ± 1.967) vs control mice (75.68 ± 2.077) (**p value = 0.0036, t value = 3.082). (**E** and **F**) Representative images of the track plot and heat map showed reduced exploratory behaviours confined to the peripheral zones. (**G**) The exploratory potential was evaluated using OFT, which revealed a significant decrease in the mean speed of ENDO (0.004154 ± 0.002586) compared with control mice (0.06107 ± 0.005146) (***p value = 0.0005, t value = 3.794). (**H**) The overall entries in the central zone were significantly reduced in ENDO (14.27 ± 1.166) vs control mice (28.36 ± 4.051) (***p value = 0.0002, t value = 4.211) and (**I**) time spent in the central area in ENDO (13.88 ± 1.936) vs control mice (29.64 ± 4.45) (***p value = 0.0005, Mann–Whitney U = 63). (**J**) While the overall entries in the peripheral zone were significantly increased in ENDO (37.5 ± 6.12) vs control mice (13 ± 1.735) (**p value = 0.0096, Mann–Whitney U = 92) and (**K**) time spent in the peripheral area in ENDO (654.8 ± 38.18) vs control mice (458.6 ± 37.16) (**p value = 0.0053, Mann–Whitney U = 85.5). Data represented as mean ± SEM and the t value and p values generated by the unpaired t test from C57BL/6J control, n = 14, and ENDO, n = 26. *p value < 0.05, **p value < 0.01,***p value < 0.001, ****p value < 0.0001 represent statistically significant values.

The anxiety parameters were calculated via EPM revealing significant reduction in entries in open arm in ENDO (12.54 ± 1.801) vs control mice (24.64 ± 1.65) (****p value < 0.0001, t value = 4.411) (Fig. 6A) and % time spent in open arm in ENDO (8.936% ± 1.819) vs control mice (20.34% ± 1.81) (***p value = 0.0003, Mann–Whitney U = 61) (Fig. 6B). As opposed to significant increase in entries in closed arms in ENDO (24.5 ± 0.8119) vs control mice (18.36 ± 1.848) (**p value = 0.003, Mann–Whitney U = 80) (Fig. 6C) and significant increase in % time spent in closed arms in ENDO (84.71 ± 1.967) vs control mice (75.68 ± 2.077) (**p value = 0.0036, t value = 3.082) (Fig. 6D). The representative images of the track plot and heat map showed reduced exploratory behaviors confined to peripheral zones (Fig. 6E and F). OFT evaluated the exploratory potential and revealed a significant decrease in mean speed of ENDO (0.004154 ± 0.002586) vs control mice (0.06107 ± 0.005146) (***p value = 0.0005, t value = 3.794) (Fig. 6G). The overall entries in central zone were significantly reduced in ENDO (14.27 ± 1.166) vs control mice (28.36 ± 4.051) (***p value = 0.0002, t value = 4.211) (Fig. 6H) and time spent in central area in ENDO (13.88 ± 1.936) vs control mice (29.64 ± 4.45) (***p value = 0.0005, Mann–Whitney U = 63) (Fig. 6I). While the overall entries in peripheral zone were significantly increased in ENDO (37.5 ± 6.12) vs control mice (13 ± 1.735) (**p value = 0.0096, Mann–Whitney U = 92) (Fig. 6J) and time spent in peripheral area in ENDO (654.8 ± 38.18) vs control mice (458.6 ± 37.16) (**p value = 0.0053, Mann–Whitney U = 85.5) (Fig. 6K). On further evaluation, the freezing episodes, total freezing time, immobility episodes, and total immobile time showed a significant increase in ENDO mice compared with controls. At the same time, the number of line crossings between zones was significantly lower in ENDO mice (Table 2). Data represented as mean ± SEM and the t value and p values generated by the unpaired t test from C57BL/6J control, n = 14, and ENDO, n = 26. *p value < 0.05, **p value < 0.01, ***p value < 0.001, ****p value < 0.0001 represent statistically significant values.

**Table 2:** OFT parameters.

Open field test parameters	Control (Mean ± SD)	ENDO (Mean ± SD)
1. Freezing episodes	3.42 ± 2.61	12.38 ± 11.14 **
2. Freezing time	11.57 ± 8.95	53.8 ± 39.15 ***
3. Immobility episodes	47.35 ± 14.22	65.42 ± 13.61 ***
4. Immobility time	247.52 ± 94.36	366.03 ± 157.5 *
5. Line crossing between zones	299.42 ± 95.31	223.08 ± 93.31 *

### MB and digging positively correlate with other non-evoked tests

**Fig. 7:**
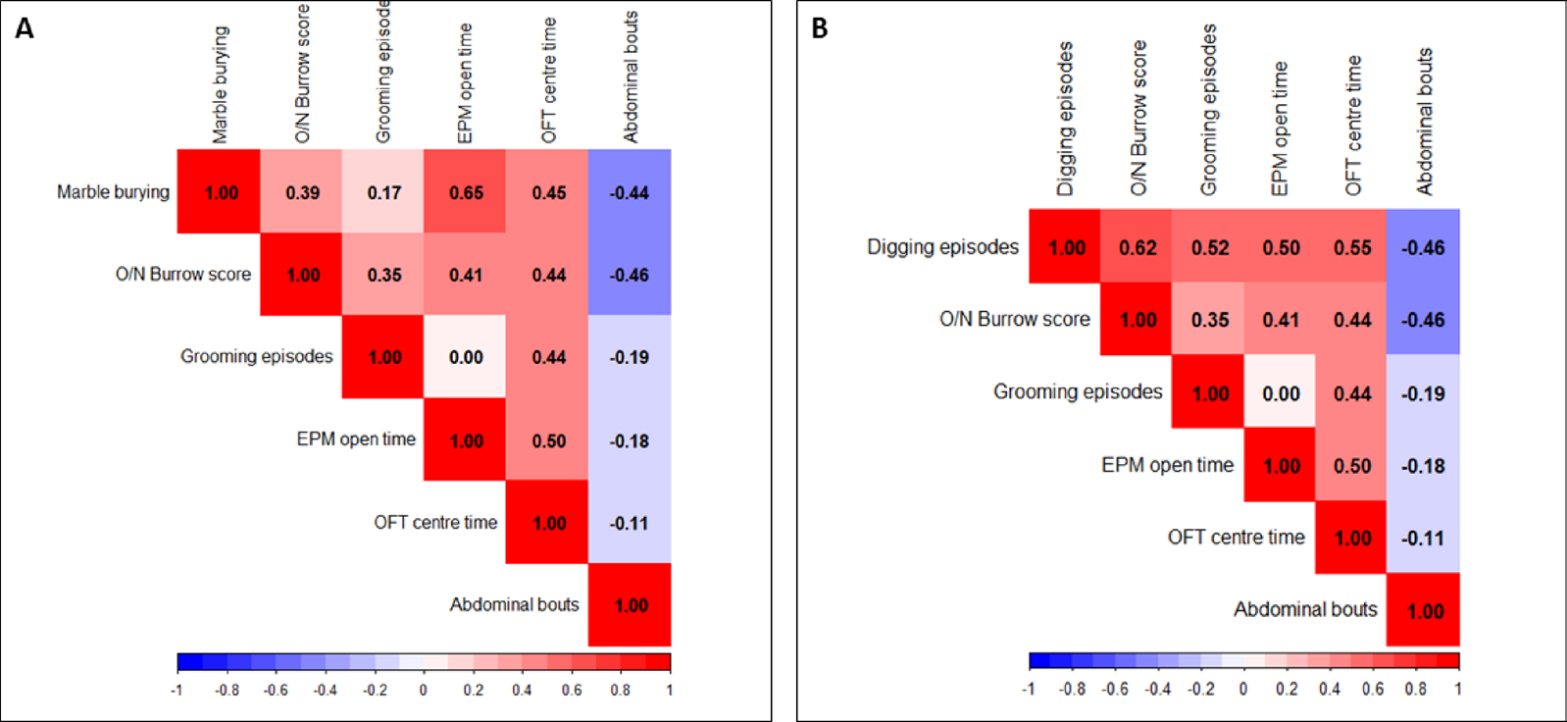
MB and Digging positively correlate with other non-evoked tests. The marble buried capacity in ENDO and control mice was correlated with Overnight burrow score, grooming episodes, EPM open time, OFT centre time, and abdominal bouts. (**A**) The highest strong positive correlation of r = 0.65 was obtained for % open time in the EPM test, followed by a moderate positive correlation of r = 0.45 for centre time in OFT, and a weak positive correlation for the overnight burrow score. The lowest positive correlation was observed for grooming episodes (r = 0.17). The marble burying showed a moderate negative correlation (r = -0.44) with abdominal-directed bout behaviour, indicating an inverse relationship between diminished marble burying capacity and abdominal-directed bouts. (**B**) Further, the digging episodes in ENDO and control mice were correlated with Overnight burrow score, grooming episodes, EPM open time, OFT centre time, and abdominal bouts. The highest strong positive correlation, r = 0.62, was obtained for overnight burrow score, followed by moderate positive correlations of r = 0.55, r = 0.52, and r = 0.5 for centre time in OFT, total grooming episodes, and % open time in EPM, respectively. The digging pattern showed a moderate negative correlation (r = -0.46) for abdominal-directed bout behaviour, indicating an inverse relation of diminished digging and abdominal-directed bouts. The values in black within each square indicate the correlation.

The marble buried capacity in ENDO and control mice was correlated with the Overnight burrow score, grooming episodes, EPM open time, OFT center time, and abdominal bouts. The strongest positive correlation, r = 0.65, was observed for % open time in the EPM test, followed by a moderate positive correlation, r = 0.45, for center time in the OFT, and a weak positive correlation for overnight burrow score. The lowest positive correlation was observed for grooming episodes (r = 0.17). The MB showed a moderate negative correlation (r = -0.44) for abdominal-directed bout behavior, indicating an inverse relation of diminished MB capacity and abdominal-directed bouts (Fig. 7A). Further, the digging episodes in ENDO and control mice were correlated with Overnight burrow score, grooming episodes, EPM open time, OFT center time, and abdominal bouts. The highest strong positive correlation, r = 0.62, was obtained for overnight burrow score, followed by moderate positive correlations of r = 0.55, r = 0.52, and r = 0.5 for center time in OFT, total grooming episodes, and % open time in EPM, respectively. The digging pattern showed a moderate negative correlation (r = -0.46) for abdominal-directed bout behavior, indicating an inverse relation of diminished digging and abdominal-directed bouts (Fig. 7B). The values in black inside each square indicate the correlation value.

### Elevated mechanical and thermal hyperalgesia is a hallmark of ENDO mice

**Fig. 8:**
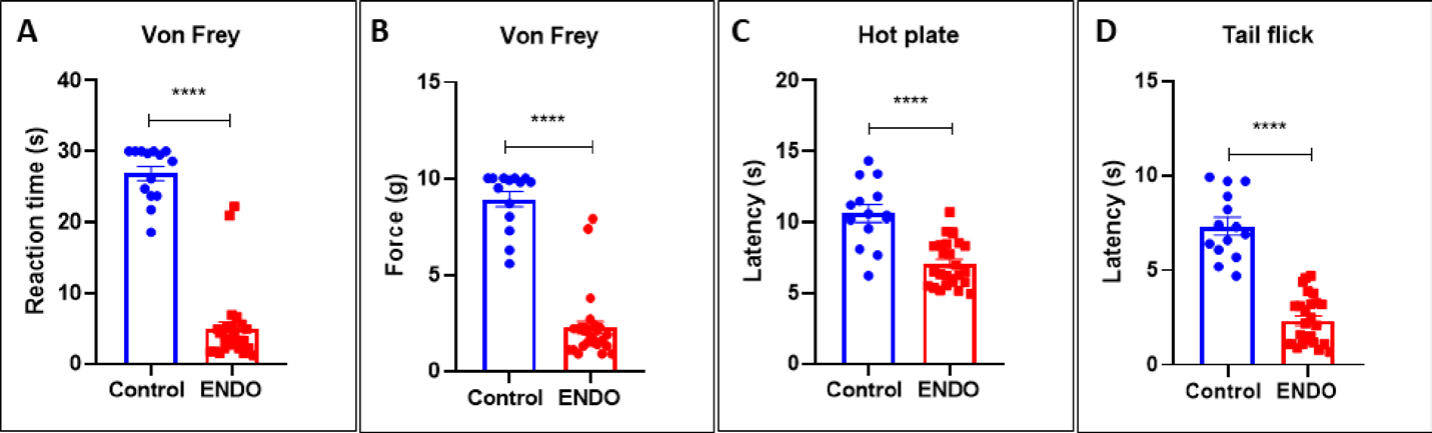
ENDO mice exhibit mechanical and thermal hyperalgesia, indicating nociception. (**A**) The mechanical hyperalgesia measure using Von Frey revealed significant reduction in reaction time in ENDO (4.927 ± 1.011) vs control mice (26.88 ± 1.015) (****p value < 0.0001, Mann–Whitney U = 3) and (**B**) reaction to force in ENDO (2.262 ± 0.337) vs control mice (8.921 ± 0.4066) (****p value < 0.0001, Mann–Whitney U = 6). (**C**) The thermal hyperalgesia measure using hot plate test revealed significant reduction in reaction time in ENDO (7.088 ± 0.3149) vs control mice (10.62 ± 0.6073) (****p value < 0.0001, Mann–Whitney U = 41) and (**D**) reduced reaction time to IR in tail flick test in ENDO (2.342 ± 0.2514) vs control mice (7.336 ± 0.4586) (****p value < 0.0001, Mann–Whitney U = 0.5). Data represented as mean ± SEM and the t value and p values generated by the unpaired t test from C57BL/6J control, n = 14, and ENDO, n = 26. ****p value < 0.0001 represents statistically significant values.

The mechanical hyperalgesia measure using Von Frey revealed significant reduction in reaction time in ENDO (4.927 ± 1.011) vs control mice (26.88 ± 1.015) (****p value < 0.0001, Mann–Whitney U = 3) (Fig. 8A) and reaction to force in ENDO (2.262 ± 0.337) vs control mice (8.921 ± 0.4066) (****p value < 0.0001, Mann–Whitney U = 6) (Fig. 8B). The thermal hyperalgesia measure using the hot plate test revealed a significant reduction in reaction time in ENDO (7.088 ± 0.3149) vs control mice (10.62 ± 0.6073) (****p value < 0.0001, Mann–Whitney U = 41) (Fig. 8C). On further evaluation using the Hargreaves test, reduced reaction time to IR in the tail flick test in ENDO (2.342 ± 0.2514) vs control mice (7.336 ± 0.4586) (****p value < 0.0001, Mann–Whitney U = 0.5) (Fig. 8D). Data represented as mean ± SEM and the t value and p values generated by the unpaired t test from C57BL/6J control, n = 14, and ENDO, n = 26. *p value < 0.05, **p value < 0.01, ****p value < 0.0001 represent statistically significant values.

### MB and digging positively correlate with evoked tests

**Fig. 9:**
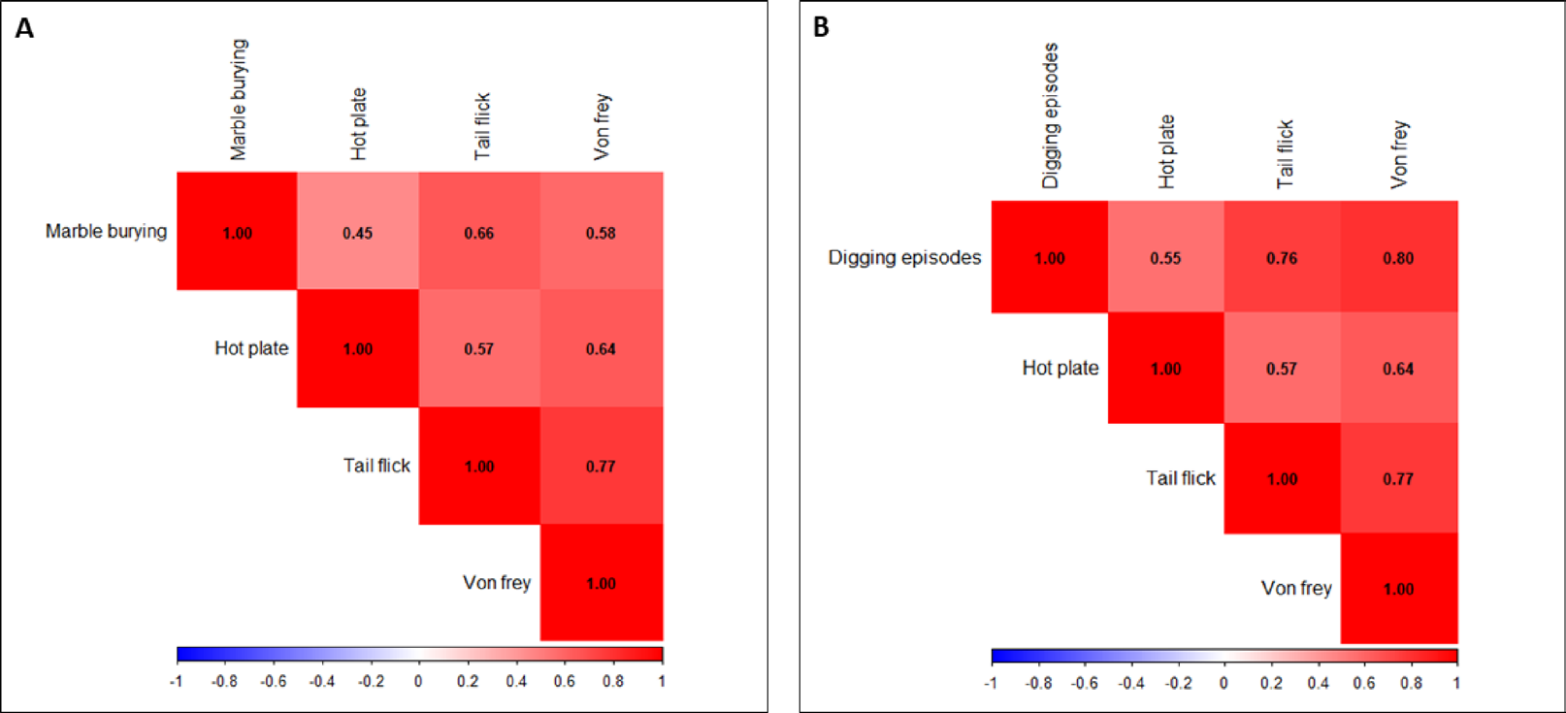
MB and Digging positively correlate with evoked tests. (**A**) The marble buried capacity in ENDO and control mice was correlated with latency to react in seconds using the hot plate, tail flick, and von Frey apparatus. The highest strong positive correlation of r = 0.66 was obtained for the tail flick test, followed by a moderate positive correlation of r = 0.58 for the Von Frey, and a moderate positive correlation of r = 0.45 for the hot plate test. (**B**) Further, the digging episodes in ENDO and control mice were correlated with latency to react in seconds using the hot plate, tail flick, and von Frey apparatus. A robust positive correlation of r = 0.8 was obtained for Von Frey, followed by a relatively strong positive correlation r = 0.76 for tail flick, and a moderate positive correlation was observed for hot plate (r = 0.55). The values in black within each square indicate the correlation.

The MB capacity in ENDO and control mice was correlated with latency to react in seconds using the hot plate, tail flick, and von Frey apparatus. The highest strong positive correlation of r = 0.66 was obtained for the tail flick test, followed by a moderate positive correlation r = 0.58 for Von Frey and a moderate positive correlation r = 0.45 for the hot plate test (Fig. 9A). Further, the digging episodes in ENDO and control mice were correlated with latency to react in seconds using the hot plate, tail flick, and von Frey apparatus. A robust positive correlation of r = 0.8 was obtained for Von Frey, followed by a relatively strong positive correlation r = 0.76 for tail flick, and a moderate positive correlation was observed for hot plate (r = 0.55) (Fig. 9B). The values in black inside each square indicate the correlation value.

## Discussion

Over the years, the paradigm of research has shifted, addressing the debilitating pain perception and reduced quality of life in women with Endometriosis. Nonetheless, the research still lacks an understanding of the multi-model pain paradigm, leading to a failure in comprehending ENDO^27^. The use of the mouse model to study ENDO has gained significant importance, as it has become easier to study many behavioral nuances that are often missed in women with ENDO^28^. Nevertheless, some models focus solely on stimulus-based pain perception in mice, thereby overlooking subtle, innate, and instinctive behaviors in ENDO models^29^. The employment of non-evoked assays aids in a better understanding of ethological parameters and helps bridge the knowledge gap regarding pain in ENDO^30^.

Successful characterization of lesion development is key in the mouse model of endometriosis, often done through lesion histology and macrophage population^31,32^. We employed a non-surgical method to induce endometriosis using donor uterine fragments, yielding lesions in 31 ENDO mice (88.57% successful model generation). The peritoneal macrophage plays a critical role in the disease progression of endometriosis^33^. The peritoneal fluid evaluation from ENDO mice also highlights elevated levels of M2 macrophages, indicating endometriosis. Further, histology of the lesion confirmed typical features of endometriotic lesions, including glandular epithelium, infiltrating stromal cells, and multinucleated immune cells. It is established that estrogen plays a key role in modulating the disease induction and progression^34,35^. Estrogen supplementation was administered to both ENDO and control mice to ensure consistent systemic estrogen exposure and minimize intergroup variability. Lastly, successful endometriosis induction was further supported by elevated serum estrogen levels in ENDO mice, indicating a systemic increase in estrogen.

The compulsive MB is an innate behavior in rodents, such as mice^20^. In our study, the ENDO mice exhibited a significantly diminished MB capacity at each time point and overall post-30 min, indicating that ENDO altered the innate and compulsive pattern in ENDO mice. Another compulsive ethological pattern is digging, which is naturally observed in rodents^16^ and can help accurately represent the human experience of pain^36^. The evaluation of digging latency in ENDO mice showed a comparison to the control mice, while the overall digging episodes were significantly reduced among ENDO mice. The deficit in digging pattern aligns with the visceral pain model, indicating ENDO alters non-evoked natural Behavioral patterns^36^. Further, the strong positive correlation of MB and digging capacity amplifies the integration of these assays for ENDO assessment. A large proportion of research focuses on lesions as the primary source of pain in women. Recent findings suggest that an aggressive lesion form may not cause severe pain, and vice versa, thereby disproving the correlation between lesion load and pain severity^37^. To investigate this in detail, correlations between MB and the number of lesions, and digging and the number of lesions in ENDO mice, were evaluated, revealing a very weak, slightly positive association for MB and a very weak, slightly negative association for digging. This further strengthens the conclusion that altered behaviour shows no strong association with lesion number or degree of lesion load.

Burrowing is an innate, naturally occurring behavior in mice, and it is reduced in inflammation-associated pain^38,39^. The ENDO mice exhibited reduced burrow score at both overnight and 2-h time points. The video recorded at the 2-h time point revealed significantly fewer active burrowing “burrow tube entries” in ENDO mice than in controls. The MB tests the ability of rodents to bury novel objects, which is often a compulsive behavior^40^. Self-directed grooming is a highly coordinated and essential behavior performed for hygiene maintenance, thermoregulation, and motivation^41^ and stress modulation^42^. The sucrose splash test revealed a reduction in grooming episodes in ENDO mice, suggesting a decline in self-grooming motivation. Greaves et al. have demonstrated increased abdominal licking and grooming behaviors in the ENDO mouse model of visceral abdominal pain^24^. Similarly, in the current study, ENDO mice show increased abdominal grooming and bout behavior, indicating abdominal visceral pain induced by ENDO.

Women with ENDO often suffer from anxiety and depression, leading to reduced quality of life^43,44^. These symptoms are frequently overlooked over other physical symptoms, but ENDO women have an increased risk of mental health conditions^45^. Li et al. 2018 have revealed that ENDO can alter pain perception, anxiety, and depression like phenomena in a mouse model^25^. Another study confirms that peritoneal endometriosis induces time-dependent depressive behavior and anxiety in the rat model^46^ and anxiety- related behavior in the mouse model of endometriosis evaluated via EPM^47^. The present study evaluated anxiety Behavioral assessment via EPM, which showed that ENDO mice remain confined to closed arms more than open arms, indicating anxiety-like behavior post ENDO induction. The mice also preferred to be confined to peripheral zones and avoided entering the central zone during OFT testing. Abdominal licking is often associated with discomfort in the pelvic area and is increased in mice experiencing pelvic pain, as seen in ENDO mouse models^48–50^. Similarly, in this study, the mice exhibited significantly more bouts and abdominal licking, confirming suspicions that ENDO leads to pelvic discomfort. The evoked and induced assays, such as the hot plate, tail flick test, and von Frey, have been widely used to assess pain and nociception in rodents. The stimulus-based pain behavior studied by Muralidharan et al. (2016) has been shown to increase nociception in rat models^25,51^. This study also evaluated mechanical and thermal stimuli to assess nociception in ENDO mice as compared to control mice. The ENDO mice showed reduced reaction latency to thermal stimuli, as measured by the hot-plate and tail-flick tests. Likewise, ENDO mice showed increased nociception to force applied via Von Frey filaments and reduced reaction latency to force. According to WERF EPHect-EM-Pain recommendations, the emphasis is on integrating non-evoked Behavioral assays to address ethologically distinguishable patterns better and on decoding the multidimensional web of pain by ENDO^6^. The positive correlation analysis shown in the corrplot graphs between MB and digging, and between non-evoked and evoked tests, further strengthens these recommendations and supports a holistic approach to Behavioral assessment in ENDO.

**Fig. 10:**
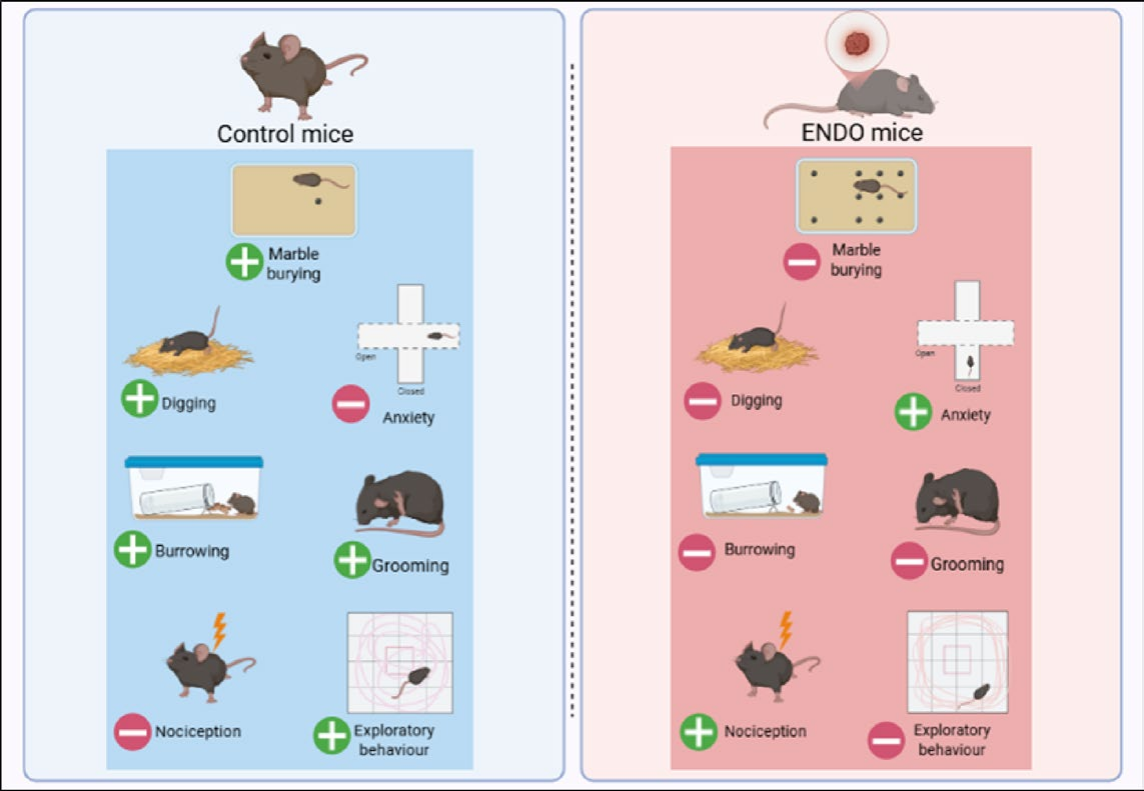
ENDO mice exhibited reduced marble burying, digging, burrowing, overall body grooming, and exploratory behaviour, but elevated anxiety and nociception.

We recognize that this research has various limitations, including the lack of a non-invasive imaging technique to confirm MB and digging behaviors and directly link these behaviors to lesion development and progression, which represents an area for future enhancement. In addition, employing specific analgesic treatments to assess the sensitivity of the MB and its role in alleviating pain would bolster its effectiveness as a biomarker. We did not explore the other molecular mechanisms underlying the altered behaviors associated with ectopic lesions. Moreover, we could only validate MB and digging behaviors in a traditional syngeneic mouse model, and further validation across different model-generation approaches will be essential to inform judgments about the general applicability of these assays. In summary, when integrating MB and digging with other Behavioral assays, this workflow will prove dependable, providing a high-confidence evaluation of EM incidence and Behavioral assessment (Fig. 10).

## Conclusion

The integration of non-evoked and evoked Behavioral assays offers a nuanced, balanced approach to decoding the web of pain in endometriosis. The employment of MB and digging capacity in a mouse model is novel for ENDO. The deep correlation analysis of these two tests with other non-evoked and evoked tests has highlighted the need for a cohesive workflow to evaluate Behavioral ethological disparity in understanding ENDO. Together, these findings establish non-evoked ethological assays as valuable complements to reflex-based nociceptive tests. Further research should focus on integrating these tests with molecular and genetic profiling to elucidate the complex pathophysiological mechanisms underlying endometriosis.

## Supplementary Information

Supplementary Information 1.

Supplementary Information 2.

Supplementary Information 3.

## Data Availability

The original contributions presented in the study are included in the article; further inquiries can be directed to the corresponding author.

## References

[1] Moradi, Y. et al. A systematic review on the prevalence of endometriosis in women. *Indian J. Med. Res.* **154**, 446–454 (2021). 35345070 10.4103/ijmr.IJMR_817_18 PMC9131783

[2] Falcone, T. & Flyckt, R. Clinical management of endometriosis. *Obstet. Gynecol.* **131**, 557 (2018). 29420391 10.1097/AOG.0000000000002469

[3] D’Hooghe, T. M. et al. Nonhuman primate models for translational research in endometriosis. *Reprod. Sci.* **16**, 152–161 (2009). 19208783 10.1177/1933719108322430

[4] Kissin, I. The development of new analgesics over the past 50 years: A lack of real breakthrough drugs. *Anesth. Analg.* **110**, 780–789 (2010). 20185657 10.1213/ANE.0b013e3181cde882

[5] Vierck, C. J., Hansson, P. T. & Yezierski, R. P. Clinical and pre-clinical pain assessment: Are we measuring the same thing?. *Pain* **135**, 7–10 (2008). 18215466 10.1016/j.pain.2007.12.008

[6] Dodds, K. N. et al. WERF endometriosis phenome and biobanking harmonisation project for experimental models in endometriosis research (EPHect-EM-Pain): methods to assess pain behaviour in rodent models of endometriosis. *Mol. Hum. Reprod.* **31**, gaaf023 (2025). 40628399 10.1093/molehr/gaaf023 PMC12237517

[7] Yezierski, R. P. & Hansson, P. Inflammatory and neuropathic pain from bench to bedside: What went wrong?. *J. Pain* **19**, 571–588 (2018). 29307749 10.1016/j.jpain.2017.12.261

[8] Schwartz, N. et al. Decreased motivation during chronic pain requires long-term depression in the nucleus accumbens. *Science* **345**, 535–542 (2014). 25082697 10.1126/science.1253994 PMC4219555

[9] De Graaff, A. A. et al. The significant effect of endometriosis on physical, mental and social wellbeing: results from an international cross-sectional survey. *Hum. Reprod. Oxf. Engl.* **28**, 2677–2685 (2013). 10.1093/humrep/det284 23847114

[10] Tejada, M. A. et al. Rodent animal models of endometriosis-associated pain: Unmet needs and resources available for improving translational research in endometriosis. *Int. J. Mol. Sci.* **24**, 2422 (2023). 36768741 10.3390/ijms24032422 PMC9917069

[11] Tejada, M. A. et al. Rodent animal models of endometriosis-associated pain: Unmet needs and resources available for improving translational research in endometriosis. *Int. J. Mol. Sci.* **24**, 2422 (2023). 36768741 10.3390/ijms24032422 PMC9917069

[12] Bashir, S. T. et al. Endometriosis leads to central nervous system-wide glial activation in a mouse model of endometriosis. *J. Neuroinflammation* **20**, 59 (2023). 36879305 10.1186/s12974-023-02713-0 PMC9987089

[13] Eisenach, J. C. & Rice, A. S. C. Improving preclinical development of novel interventions to treat pain: Insanity is doing the same thing over and over and expecting different results. *Anesth. Analg.* **135**, 1128–1136 (2022). 36384008 10.1213/ANE.0000000000006249 PMC9976707

[14] Sadler, K. E., Mogil, J. S. & Stucky, C. L. Innovations and advances in modelling and measuring pain in animals. *Nat. Rev. Neurosci.* **23**, 70–85 (2022). 34837072 10.1038/s41583-021-00536-7 PMC9098196

[15] Negus, S. S. Core outcome measures in preclinical assessment of candidate analgesics. *Pharmacol. Rev.* **71**, 225–266 (2019). 30898855 10.1124/pr.118.017210 PMC6448246

[16] Deacon, R. M. J. Digging and marble burying in mice: Simple methods for in vivo identification of biological impacts. *Nat. Protoc.* **1**, 122–124 (2006). 17406223 10.1038/nprot.2006.20

[17] Njung’e, K. & Handley, S. L. Evaluation of marble-burying behavior as a model of anxiety. *Pharmacol. Biochem. Behav.* **38**, 63–67 (1991). 2017455 10.1016/0091-3057(91)90590-x

[18] Deacon, R. M. J. & Rawlins, J. N. P. Hippocampal lesions, species-typical behaviours and anxiety in mice. *Behav. Brain Res.* **156**, 241–249 (2005). 15582110 10.1016/j.bbr.2004.05.027

[19] Anchan, M. et al. C57BL/6J mice best recapitulate fibrosis and inflammatory pathophysiology in syngeneic mouse model of endometriosis. *Sci. Rep.* **15**, 29024 (2025). 40781536 10.1038/s41598-025-13900-9 PMC12334603

[20] Thomas, A. et al. Marble burying reflects a repetitive and perseverative behavior more than novelty-induced anxiety. *Psychopharmacology* **204**, 361–373 (2009). 19189082 10.1007/s00213-009-1466-y PMC2899706

[21] Deacon, R. M. J. Burrowing in rodents: A sensitive method for detecting behavioral dysfunction. *Nat. Protoc.* **1**, 118–121 (2006). 17406222 10.1038/nprot.2006.19

[22] Anchan, M. et al. Burrowing behavior is a potential non-invasive proxy for lesion development in a syngeneic murine model of endometriosis. *BMC Womens Health*10.1186/s12905-025-04112-4 (2025). 41388524 10.1186/s12905-025-04112-4 PMC12822023

[23] Barrot, M. Tests and models of nociception and pain in rodents. *Neuroscience* **211**, 39–50 (2012). 22244975 10.1016/j.neuroscience.2011.12.041

[24] Greaves, E. et al. EP2 receptor antagonism reduces peripheral and central hyperalgesia in a preclinical mouse model of endometriosis. *Sci. Rep.* **7**, 44169 (2017). 28281561 10.1038/srep44169 PMC5345039

[25] Li, T. et al. Endometriosis alters brain electrophysiology, gene expression and increases pain sensitization, anxiety, and depression in female mice†. *Biol. Reprod.* **99**, 349–359 (2018). 29425272 10.1093/biolre/ioy035 PMC6692844

[26] Citterio, F., Corradini, L., Smith, R. D. & Bertorelli, R. Nociceptin attenuates opioid and γ-aminobutyric acidB receptor-mediated analgesia in the mouse tail-flick assay. *Neurosci. Lett.* **292**, 83–86 (2000). 10998554 10.1016/s0304-3940(00)01448-8

[27] Maddern, J., Grundy, L., Castro, J. & Brierley, S. M. Pain in endometriosis. *Front. Cell. Neurosci.* **14**, 590823 (2020). 33132854 10.3389/fncel.2020.590823 PMC7573391

[28] Bruner-Tran, K. L., Mokshagundam, S., Herington, J. L., Ding, T. & Osteen, K. G. Rodent models of experimental endometriosis: Identifying mechanisms of disease and therapeutic targets. *Current Women s Health Reviews* **14**, 173–188 (2018). 29861705 10.2174/1573404813666170921162041 PMC5925870

[29] Fried, N. T., Chamessian, A., Zylka, M. J. & Abdus-Saboor, I. Improving pain assessment in mice and rats with advanced videography and computational approaches. *Pain* **161**, 1420 (2020). 32102021 10.1097/j.pain.0000000000001843 PMC7302333

[30] Tejada, M. A. et al. Identification of altered evoked and non-evoked responses in a heterologous mouse model of endometriosis-associated pain. *Biomedicines* **10**, 501 (2022). 35203710 10.3390/biomedicines10020501 PMC8962432

[31] Vigano, P. et al. Time to redefine endometriosis including its pro-fibrotic nature. *Hum. Reprod. Oxf. Engl.* **33**, 347–352 (2018). 10.1093/humrep/dex354 29206943

[32] Hogg, C., Horne, A. W. & Greaves, E. Endometriosis-associated macrophages: Origin, phenotype, and function. *Front. Endocrinol.* **11**, 7 (2020). 10.3389/fendo.2020.00007 PMC6989423 32038499

[33] Ramírez-Pavez, T. N. et al. The Role of Peritoneal Macrophages in Endometriosis. *Int. J. Mol. Sci.* **22**, 10792 (2021). 34639133 10.3390/ijms221910792 PMC8509388

[34] Giudice, L. C. & Kao, L. C. Endometriosis. *Lancet* **364**, 1789–1799 (2004). 15541453 10.1016/S0140-6736(04)17403-5

[35] Zondervan, K. T., Becker, C. M. & Missmer, S. A. Endometriosis. *N. Engl. J. Med.* **382**, 1244–1256 (2020). 32212520 10.1056/NEJMra1810764

[36] Pattison, L. A. et al. Digging deeper into pain: an ethological behavior assay correlating well-being in mice with human pain experience. *Pain* **165**, 1761 (2024). 38452214 10.1097/j.pain.0000000000003190 PMC11247454

[37] Vercellini, P. et al. Association between endometriosis stage, lesion type, patient characteristics and severity of pelvic pain symptoms: A multivariate analysis of over 1000 patients. *Hum. Reprod. (Oxf. Engl.).* **22**, 266–271 (2007). 10.1093/humrep/del339 16936305

[38] Andrews, N. et al. Spontaneous burrowing behaviour in the rat is reduced by peripheral nerve injury or inflammation associated pain. *Eur. J. Pain* **16**, 485–495 (2012). 22396078 10.1016/j.ejpain.2011.07.012

[39] Shepherd, A. J., Cloud, M. E., Cao, Y.-Q. & Mohapatra, D. P. Deficits in burrowing behaviors are associated with mouse models of neuropathic but not inflammatory pain or migraine. *Front. Behav. Neurosci.* **12**, 124 (2018). 30002622 10.3389/fnbeh.2018.00124 PMC6031738

[40] Angoa-Pérez, M., Kane, M. J., Briggs, D. I., Francescutti, D. M. & Kuhn, D. M. Marble burying and nestlet shredding as tests of repetitive, compulsive-like behaviors in mice. *J. Vis. Exp. JoVE.* **82**, 50978. 10.3791/50978 (2013). 10.3791/50978 PMC4108161 24429507

[41] Smolinsky, A. N., Bergner, C. L., LaPorte, J. L. & Kalueff, A. V. Analysis of grooming behavior and its utility in studying animal stress, anxiety, and depression. In *Mood and Anxiety Related Phenotypes in Mice: Characterization Using Behavioral Tests* (ed. Gould, T. D.) 21–36 (Humana Press, 2009). 10.1007/978-1-60761-303-9_2.

[42] Kalueff, A. V. et al. Neurobiology of rodent self-grooming and its value for translational neuroscience. *Nat. Rev. Neurosci.* **17**, 45–59 (2016). 26675822 10.1038/nrn.2015.8 PMC4840777

[43] Hernandes, C. et al. Microbiome Profile of Deep Endometriosis Patients: Comparison of Vaginal Fluid. *Endometrium and Lesion. Diagnostics* **10**, 163 (2020). 32192080 10.3390/diagnostics10030163 PMC7151170

[44] Szypłowska, M., Tarkowski, R. & Kułak, K. The impact of endometriosis on depressive and anxiety symptoms and quality of life: a systematic review. *Front. Public Health* **11**, 1230303 (2023). 37744486 10.3389/fpubh.2023.1230303 PMC10512020

[45] Thiel, P. S. et al. Endometriosis and mental health: A population-based cohort study. *Am. J. Obstet. Gynecol.* **230**, 649.e1-649.e19 (2024). 38307469 10.1016/j.ajog.2024.01.023

[46] Filho, P. W. L. L. et al. Peritoneal endometriosis induces time-related depressive- and anxiety-like alterations in female rats: involvement of hippocampal pro-oxidative and BDNF alterations. *Metab. Brain Dis.* **34**, 909–925 (2019). 30798429 10.1007/s11011-019-00397-1

[47] Nunez-Badinez, P. et al. Anxiety-related behaviors without observation of generalized pain in a mouse model of endometriosis. *Front. Behav. Neurosci.* **17**, 1118598 (2023). 36844654 10.3389/fnbeh.2023.1118598 PMC9947402

[48] Fattori, V. et al. Nociceptor-to-macrophage communication through CGRP/RAMP1 signaling drives endometriosis-associated pain and lesion growth in mice. *Sci. Transl. Med.* **16**, eadk8230 (2024). 39504351 10.1126/scitranslmed.adk8230

[49] Zaninelli, T. H. et al. Targeting NGF but not VEGFR1 or BDNF signaling reduces endometriosis-associated pain in mice. *J. Adv. Res.* **73**, 593–605 (2025). 39142441 10.1016/j.jare.2024.08.017 PMC12225929

[50] Fattori, V. et al. Nonsurgical mouse model of endometriosis-associated pain that responds to clinically active drugs. *Pain* **161**, 1321 (2020). 32132396 10.1097/j.pain.0000000000001832

[51] Muralidharan, A. et al. Comparison of burrowing and stimuli-evoked pain behaviors as end-points in rat models of inflammatory pain and peripheral neuropathic pain. *Front. Behav. Neurosci.* **10**, 88 (2016). 27242458 10.3389/fnbeh.2016.00088 PMC4862327

