# Supplementary material for: Behavioral attenuation of marble burying and digging mirrors evoked and non-evoked phenotypes in the endometriosis mouse model
Source: Sci Rep. 2026 Feb 20;16:10007. doi: 10.1038/s41598-026-40662-9 (PMC13022329; doi:10.1038/s41598-026-40662-9)
Supplement: Supplementary file 1 — Supplementary Information 1. [file 41598_2026_40662_MOESM1_ESM.docx]

| **Open field test parameters** | **Control (Mean ± SD)** | **ENDO (Mean ± SD)** |
| --- | --- | --- |
| 1. Freezing episodes | 3.42 ± 2.61 | 12.38 ± 11.14 ** |
| 2. Freezing time | 11.57 ± 8.95 | 53.8 ± 39.15 *** |
| 3. Immobility episodes | 47.35 ± 14.22 | 65.42 ± 13.61 *** |
| 4. Immobility time | 247.52 ± 94.36 | 366.03 ± 157.5 * |
| 5. Line crossing between zones | 299.42 ± 95.31 | 223.08 ± 93.31 * |

Table 1: OFT supplementary parameters.

ARRIVE guidelines checklist:

- Species and strain: Adult female C57BL/6j mice (Mus musculus) were used in this study.
- Sex: Adult female mice.
- Age and weight: Mice of age 8-9 weeks at the start of experiments and weighed 20-30 g.
- Source and health status: Animals were procured from the Central Animal Research Facility at Manipal Academy of Higher Education
- Housing and husbandry: Mice were housed in groups 4 per cage under standard laboratory conditions (12 h light/dark cycle, temperature 23±2 °C, humidity 50–55%), with ad libitum access to food and water.
- Acclimatization: Animals were acclimatized to the housing environment for at least 3 days prior to experimental procedures.
- Experimental groups: Mice were randomly assigned to experimental groups:

1. Control group (n = 14)
2. Endometriosis (ENDO) group (n = 26)

- Inclusion and exclusion criteria: Animals were included if they showed no signs of infection or distress unrelated to the experimental condition.
- Randomization: Animals were randomly allocated as donors, control and ENDO groups.
- Blinding: Experimenters were blinded to group allocation during behavioral testing and data analysis.
- Ethical approval: The study was approved by the Institutional Animal Ethics Committee at Kasturba Medical College, Manipal (Approval Number: C57BL/6j- IAEC/KMC/20/2025).
- Welfare and monitoring: Animals were monitored daily for general health, body weight, and signs of pain or distress.

Table 2: Weight of control animals

| **Control Weight** | | | | | | | | | | | | | | | |
| --- | --- | --- | --- | --- | --- | --- | --- | --- | --- | --- | --- | --- | --- | --- | --- |
|  |  | **1** | **2** | **3** | **4** | **5** | **6** | **7** | **8** | **9** | **10** | **11** | **12** | **13** | **14** |
| **Day 1** |  | 23.6 | 21.2 | 25.4 | 28.6 | 26.5 | 23.5 | 27.8 | 26.5 | 20.6 | 28 | 22.1 | 29 | 28.6 | 29.8 |
| **Day 2** |  | 24.5 | 21.3 | 25.3 | 28.06 | 25.08 | 23.6 | 27.2 | 25.1 | 20.1 | 28 | 22.3 | 29.3 | 26.6 | 30 |
| **Day 3** |  | 24 | 20.9 | 25 | 27.08 | 26.3 | 25 | 27 | 25.06 | 20.1 | 27.8 | 22.08 | 29.8 | 27.6 | 30.1 |
| **Day 4** |  | 24.8 | 22.1 | 26.1 | 27.08 | 26.2 | 24.7 | 27.04 | 25.8 | 20 | 27.9 | 21.5 | 29.5 | 27 | 30.9 |
| **Day 5** |  | 25.3 | 22.3 | 25.8 | 27 | 26 | 25 | 28 | 26.3 | 20.3 | 29.2 | 21.7 | 29.5 | 27.4 | 30.2 |
| **Day 6** |  | 24.9 | 21.9 | 26 | 26.6 | 26.6 | 25.8 | 27.6 | 26.5 | 20.4 | 28.5 | 22.2 | 29.5 | 27 | 30.6 |
| **Day 7** |  | 24 | 22 | 26.1 | 26.7 | 26.4 | 25.9 | 27.3 | 26.5 | 20.4 | 29 | 22.7 | 30.1 | 28.8 | 31.9 |
| **Day 8** |  | 24.3 | 22.1 | 25.8 | 27.1 | 27 | 26 | 27.3 | 26.8 | 20.6 | 29.3 | 22.7 | 30.3 | 29 | 32 |
| **Day 9** |  | 24.4 | 22.3 | 26.2 | 27 | 27.2 | 26.1 | 28 | 26.3 | 20.8 | 29.5 | 23 | 30 | 29.1 | 31.8 |
| **Day 10** |  | 25 | 22.2 | 26.3 | 27.1 | 27 | 26.4 | 28.1 | 26.4 | 21 | 30.1 | 23.2 | 29.8 | 29.4 | 32.1 |
| **Day 11** |  | 25.1 | 23 | 26 | 27.3 | 27.3 | 26.9 | 28.4 | 26.8 | 21.3 | 30.1 | 23.5 | 30.1 | 29.4 | 31.7 |
| **Day 12** |  | 25.3 | 23.2 | 26.4 | 27.5 | 27.4 | 26.6 | 28 | 27 | 21.4 | 30.3 | 23.5 | 30.2 | 29.5 | 31.6 |

Table 3: Weight of ENDO animal (part 1)

| **ENDO weight (Animals 1-14)** | | | | | | | | | | | | | | |
| --- | --- | --- | --- | --- | --- | --- | --- | --- | --- | --- | --- | --- | --- | --- |
|  | **1** | **2** | **3** | **4** | **5** | **6** | **7** | **8** | **9** | **10** | **11** | **12** | **13** | **14** |
| **Day 1** | 24.3 | 23.3 | 27.9 | 25.6 | 22.1 | 21 | 20.8 | 28.3 | 22.3 | 27.3 | 23.7 | 28.1 | 29.9 | 23.6 |
| **Day 2** | 24.4 | 23.5 | 27.8 | 25.8 | 22.3 | 21.1 | 21 | 28.2 | 22.2 | 28.2 | 24.6 | 28.8 | 30.3 | 24 |
| **Day 3** | 24.7 | 23.9 | 27.9 | 25.8 | 23.1 | 22.3 | 21.3 | 28.4 | 22 | 29.7 | 25.8 | 29.9 | 30.3 | 25.1 |
| **Day 4** | 25.4 | 23.8 | 27 | 25.6 | 23 | 22.2 | 21.3 | 28.3 | 22.8 | 30.2 | 26.5 | 32.1 | 32.3 | 23.7 |
| **Day 5** | 26.5 | 24.5 | 27.2 | 25.7 | 22.9 | 22.4 | 21.5 | 28.5 | 22 | 30 | 26.5 | 31.9 | 32.4 | 23.5 |
| **Day 6** | 26.8 | 24.8 | 27.8 | 26.2 | 23.1 | 22.3 | 21.5 | 28.7 | 21.8 | 30.3 | 26.8 | 31.2 | 32.8 | 23.5 |
| **Day 7** | 26.8 | 24.6 | 27.9 | 26.9 | 23.4 | 22.5 | 21 | 28.9 | 21.3 | 29.8 | 26 | 30.9 | 30.6 | 25.2 |
| **Day 8** | 27.2 | 24.5 | 27.6 | 26.9 | 23.2 | 22.6 | 21.2 | 29.4 | 22.2 | 29.9 | 26.2 | 29.9 | 30.8 | 25.8 |
| **Day 9** | 27.3 | 24.6 | 28 | 27.2 | 23.6 | 22.8 | 21.3 | 29.8 | 22 | 29.1 | 25.9 | 30.2 | 31 | 25.4 |
| **Day 10** | 27.3 | 24.7 | 28.1 | 28 | 23.5 | 22.8 | 22 | 29.8 | 22.2 | 29 | 26.1 | 30.3 | 30.9 | 25.6 |
| **Day 11** | 27.5 | 25.1 | 28.2 | 27.9 | 23.8 | 23.1 | 22.4 | 30.1 | 22.5 | 28.8 | 26.3 | 30 | 30.6 | 25.6 |
| **Day 12** | 27.8 | 25 | 28.3 | 28 | 23.7 | 23.2 | 22.5 | 30.2 | 22.6 | 29.3 | 27 | 30.1 | 30.4 | 26.3 |

Table 4: Weight of ENDO animal (part 2)

| **ENDO weight (Animals 15-26)** | | | | | | | | | | | | |
| --- | --- | --- | --- | --- | --- | --- | --- | --- | --- | --- | --- | --- |
|  | **15** | **16** | **17** | **18** | **19** | **20** | **21** | **22** | **23** | **24** | **25** | **26** |
| **Day 1** | 27.1 | 27.4 | 24.6 | 23.4 | 27.2 | 28.5 | 24.6 | 22 | 27.9 | 29.2 | 29.8 | 29.7 |
| **Day 2** | 28 | 28.1 | 25.2 | 24.1 | 28.1 | 28.3 | 24.6 | 22.5 | 28.4 | 29.3 | 29.8 | 30.7 |
| **Day 3** | 28.1 | 29.2 | 26.1 | 24.8 | 27 | 28.3 | 23.8 | 21.5 | 27.3 | 28.7 | 29 | 30.4 |
| **Day 4** | 25.8 | 27.6 | 23.8 | 24.4 | 27.1 | 28 | 22 | 21.5 | 26 | 28 | 29 | 30 |
| **Day 5** | 25 | 27.2 | 22.8 | 24 | 26.9 | 28.1 | 22.5 | 22.3 | 26.8 | 28.4 | 29.4 | 29.8 |
| **Day 6** | 24.8 | 28.1 | 22.9 | 22.2 | 26.8 | 28.5 | 22.6 | 22.8 | 26.9 | 28.5 | 29.6 | 30.1 |
| **Day 7** | 28 | 30 | 25.4 | 24 | 27 | 29 | 23.3 | 23.1 | 27.2 | 29.1 | 30 | 30.3 |
| **Day 8** | 28.1 | 29.8 | 26.2 | 25 | 27.2 | 28.8 | 23.5 | 22.9 | 27.3 | 29.3 | 29.6 | 30.3 |
| **Day 9** | 28.3 | 29.7 | 26.3 | 25.2 | 27.2 | 29.3 | 23 | 23.6 | 27.4 | 29.6 | 29.8 | 30.7 |
| **Day 10** | 28 | 30.1 | 26.4 | 25.4 | 27.5 | 29.5 | 23.8 | 23.8 | 27.4 | 30.2 | 30.3 | 31 |
| **Day 11** | 27.5 | 29.7 | 26.7 | 25.8 | 27.6 | 29.5 | 24.2 | 24.3 | 27.6 | 30.2 | 30.7 | 31.1 |
| **Day 12** | 27.5 | 30.1 | 27.1 | 26 | 28 | 30.2 | 24.5 | 24.4 | 28 | 30.5 | 30.5 | 31 |

Table 5: Non-parametric test (Mann-whitney test)

| Sr. No | Test parameter | Exact P value | Mann-Whitney score |
| --- | --- | --- | --- |
| 1 | Total marbles buried at 30 mins | <0.0001**** | 16 |
| 2 | Digging latency | 0.1183 (ns) | 129 |
| 3 | Digging episodes | <0.0001**** | 0 |
| 4 | Overnight burrow score | <0.0001**** | 8 |
| 5 | 2-hour burrow score | 0.0002*** | 56.50 |
| 6 | Active burrow entries | 0.0004*** | 62 |
| 7 | Time spent in burrow tube | 0.0637 (ns) | 116.5 |
| 8 | Latency to burrow | 0.1603 (ns) | 132 |
| 9 | Grooming latency | 0.8610 (ns) | 175.5 |
| 10 | Grooming episodes | 0.0241* | 103 |
| 11 | Abdominal directed licking | <0.0001**** | 21.50 |
| 12 | EPM entries in open | 0.0002*** | 57.50 |
| 13 | % time spent in open | 0.0003*** | 61 |
| 14 | EPM entries in closed | 0.0030** | 80 |
| 15 | % time spent in closed | 0.0012** | 102 |
| 16 | Mean speed | 0.0005*** | 65 |
| 17 | Entries in central area | 0.0015** | 73.50 |
| 18 | Time spent in central area | 0.0005*** | 63 |
| 19 | Entries in peripheral area | 0.0096** | 92 |
| 20 | Time spent in peripheral area | 0.0053** | 85.50 |
| 21 | Von Frey Reaction time | <0.0001**** | 3 |
| 22 | Von frey Force | <0.0001**** | 6 |
| 23 | Hot plate | <0.0001**** | 41 |
| 24 | Tail flick | <0.0001**** | 0.5 |
